# Cannabis Use Among Older Adults

**DOI:** 10.1001/jamanetworkopen.2025.10173

**Published:** 2025-05-14

**Authors:** Vira Pravosud, Emily Lum, Marzieh Vali, Beth E. Cohen, Katherine J. Hoggatt, Amy L. Byers, Peter C. Austin, Louise C. Walter, Deborah Hasin, Tauheed Zaman, Salomeh Keyhani

**Affiliations:** 1 Center for Data to Discovery and Delivery Innovation, San Francisco Veterans Affairs (VA) Health Care System, San Francisco, California; 2 Northern California Institute for Research and Education, San Francisco; 3 Center for Tobacco Control Research and Education, Cardiovascular Research Institute, University of California, San Francisco; 4 School of Medicine, University of California, San Francisco; 5 Division of Geriatrics, San Francisco VA Health Care System, San Francisco, California; 6 Division of Geriatrics, Department of Medicine, University of California, San Francisco; 7 Department of Psychiatry and Behavioral Sciences, University of California, San Francisco; 8 Institute of Health Policy, Management and Evaluation, University of Toronto, Toronto, Ontario, Canada; 9 Department of Psychiatry, Columbia University Medical Center, New York, New York; 10 Addiction Recovery and Treatments Services, San Francisco VA Health Care System, San Francisco VA Medical Center, San Francisco, California

## Abstract

**Importance:**

Little is known about patterns (forms, frequency, and reasons) and factors associated with cannabis use in older veterans (aged ≥65 years).

**Objective:**

To examine factors associated with past 30-day cannabis use and cannabis use disorder (CUD) in older veterans.

**Design, Setting, and Participants:**

In this cross-sectional study, community-dwelling adults aged 65 to 84 years who used Veterans Health Administration care were interviewed between February 5, 2020, and August 29, 2023.

**Exposure:**

Sociodemographic, behavioral, and health-related characteristics.

**Main Outcomes and Measures:**

Past 30-day cannabis use (smoking, vaping, dabbing, or edibles) and any CUD (≥2 criteria based on *Diagnostic and Statistical Manual of Mental Disorders* [Fifth Edition]) were assessed using weighted multivariable logistic regressions.

**Results:**

Of the 4503 participants (weighted mean age, 73.3 years [95% CI, 73.0-73.5 years]; 85.4% [95% CI, 83.6%-87.2%] men), 58.2% (95% CI, 55.3%-61.0%) had ever used cannabis, 28.9% (95% CI, 26.0%-31.8%) of whom reported using cannabis for medical reasons, most commonly for pain (56.4%; 95% CI, 50.9%-61.9%), mood or mental health (18.4%; 95% CI, 14.7%-22.1%), and sleep (16.0%; 95% CI, 11.9%-20.0%). More than 1 in 10 reported past 30-day cannabis use (10.3%; 95% CI, 8.9%-11.7%), with 52.4% (95% CI, 45.4%-59.4%) of these using cannabis for 20 days or more; smoking (72.4%; 95% CI, 65.4%-79.3%) and edibles (36.9%; 95% CI, 29.8%-43.9%) were the most common forms of use. Characteristics associated with past 30-day use included younger age (65-75 years), economic hardship, tobacco and illicit drug use, and residing in a state with recreationally legal cannabis. Among those with past 30-day cannabis use, 36.3% (95% CI, 30.1%-42.6%) screened positive for CUD, with higher odds among younger respondents, those reporting anxiety, those with 1 or more deficits in activities of daily living, those with illicit drug use, those with frequent cannabis use, and those using cannabis recreationally. Past 30-day inhaled cannabis use, compared with edibles only, was associated with increased odds of any CUD (adjusted odds ratio, 3.56; 95% CI, 1.12-11.26).

**Conclusions and Relevance:**

In this cross-sectional study of cannabis use in older veterans, use was common, and more than one-third who used in the past 30 days had any CUD. The prevalence of past 30-day cannabis use was close to tobacco use prevalence, and risk factors for cannabis use were similar to those observed in other populations. Frequent and inhaled cannabis use was associated with higher odds of any CUD. Routine health screening for cannabis use in Veterans Health Administration clinical settings is necessary to identify older adults with cannabis use.

## Key Points

**Question:**

What are the prevalence and correlates of cannabis use and cannabis use disorder (CUD) among older adults?

**Findings:**

In this cross-sectional study of 4503 respondents aged 65 to 84 years from the Veterans Health Administration (2020-2023), 10% reported past 30-day cannabis use, 36% of whom had CUD. Odds for CUD were higher among younger respondents, those reporting anxiety, those with 1 or more deficits in activities of daily living, those with past month illicit drug use, and those with frequent, inhaled, or recreational cannabis use.

**Meaning:**

Asking about cannabis use as part of routine health behavior screening in Veterans Health Administration clinical settings may help identify older adults with cannabis use or CUD.

## Teaser

This cross-sectional study examines the prevalence of cannabis use and cannabis use disorder among older veterans in the US.

## Introduction

Cannabis use has been increasing in the US,^1,2,3^ yet little is known about patterns and correlates of current cannabis use and cannabis use disorder (CUD) in older adults.^4,5^ Previous studies have suggested an increase in cannabis use among older people,^4,5,6,7,8^ with less than 1% (ranging from 0.4%^6,8^ to 0.7%^7^) of adults 65 years or older reporting past-year cannabis use in 2005 to 2006 compared with 8.4% in 2022.^9^ Smoking is the most common form of cannabis use among those 60 years or older^4,5^ and is perceived as less harmful than tobacco smoking.^6,10^ Expansion of state-directed legalization of medical and recreational cannabis, which leads to greater accessibility of cannabis products, has contributed to higher rates of cannabis use^1,11,12,13,14,15^ and CUD.^13,16^ Between 2017 and 2022, there was a significant increase in CUD-related encounters among Medicare beneficiaries 65 years or older, with the highest rates in states where recreational and medical cannabis was legal.^17^ Despite older adults generally holding more negative beliefs about cannabis, legalization might have shifted cannabis perceptions toward its social acceptance among this age group.^18^ Older people use cannabis for medical reasons, such as management of pain, mental health, and sleep, which can influence their risk perception and increase the frequency of use.^19,20^

Favorable views and increasing frequency of cannabis use among older adults warrant more research on patterns of consumption and correlates of CUD among this population. Older people are generally at higher risks for impairment of activities of daily living (ADLs),^21^ frailty and associated falls,^22^ as well as hospitalizations and mortality^23^ and thus may be particularly susceptible to adverse effects of cannabis, even if used for therapeutic purposes.^24,25^ Cannabis use increases the risk of neuropsychiatric disorders,^26,27,28,29^ respiratory symptoms,^30^ and cardiovascular outcomes^31,32,33,34,35^ that remain the leading causes of death in older adults.^23^ Another negative consequence of cannabis use is the risk of CUD, which has become one of the most prevalent substance use disorders in the US.^3^ Data from the early 1990s to mid-2000s indicate that approximately 20% to 30% of those who use cannabis may develop CUD,^36^ with higher risks for people who use cannabis daily.^37,38^ However, these data rely on reports largely from younger populations. In recent years, frequent cannabis use has increased in the general US population 12 years and older,^39^ but the frequency of current cannabis use and its association with CUD in older adults remains unclear.

Although some previous studies have examined cannabis use and CUD in middle to late life,^7,40,41,42^ to our knowledge, no recent study has focused on detailing the patterns and correlates of current cannabis use and CUD in adults 65 years or older at a national level. Older veterans are another understudied population in cannabis research. Unlike younger age groups (18-25 years), veterans 65 years or older are less likely to use recreational cannabis,^43,44^ are more likely to use medicinal cannabis recommended by a health care professional,^45^ and report use for pain management, insomnia, and mental health (eg, posttraumatic stress disorder [PTSD]).^19,20,46^ Overall, cannabis use and CUD among US veterans 18 years or older, including among patients of the Veterans Health Administration (VHA), have been increasing since the early 2000s,^45,47^ especially in states that have legalized cannabis.^16,43,47^ During the early and mid-2000s, past-year CUD estimates were 1.38% among VHA patients^16^ and 1.8% among US veterans in general.^43^ The most recent 2020 data of US veterans aged 22 to 99 years (mean [SD] age, 62.2 [15.7] years) showed a rate of 2.7% for past 6-month CUD diagnoses.^48^ In VHA patients 65 years or older, CUD prevalence has disproportionately increased between 2016 and 2019 among veterans with psychiatric disorders (from 1.3% to 2.0%) vs those without^49^ and among those with chronic or severe pain (from 0.6% to 1.0%) vs those without.^46^ However, rates of CUD among older veterans who engage in cannabis use are unknown.

The current Veterans Affairs (VA) Cannabis and Aging Study is the first analysis of a cohort of adults 65 years or older who have answered detailed survey questions on cannabis use and CUD. We used cross-sectional data from the baseline interview of this cohort to characterize cannabis use in late life, describing forms, frequency, reasons for cannabis use, and prevalence and correlates of CUD among a national sample of older adults who used the VA health care system. Prior research of older adults among veteran^16,46,49^ and nonveteran^17^ populations in health care settings provides data on CUD prevalence based on clinical diagnoses, which likely represent more severe CUD cases,^50,51^ whereas rates of mild CUD may remain underreported.^52^ Although the use of a VHA cohort in our study presents challenges for generalizability to other populations,^53^ this work provides important data on cannabis use in older veterans and offers benchmark data regarding patterns and correlates among the general population of adults 65 years or older—an age group that has been underrepresented in cannabis research.^37,54^

## Methods

### Study Design and Sample

**Figure 1.**
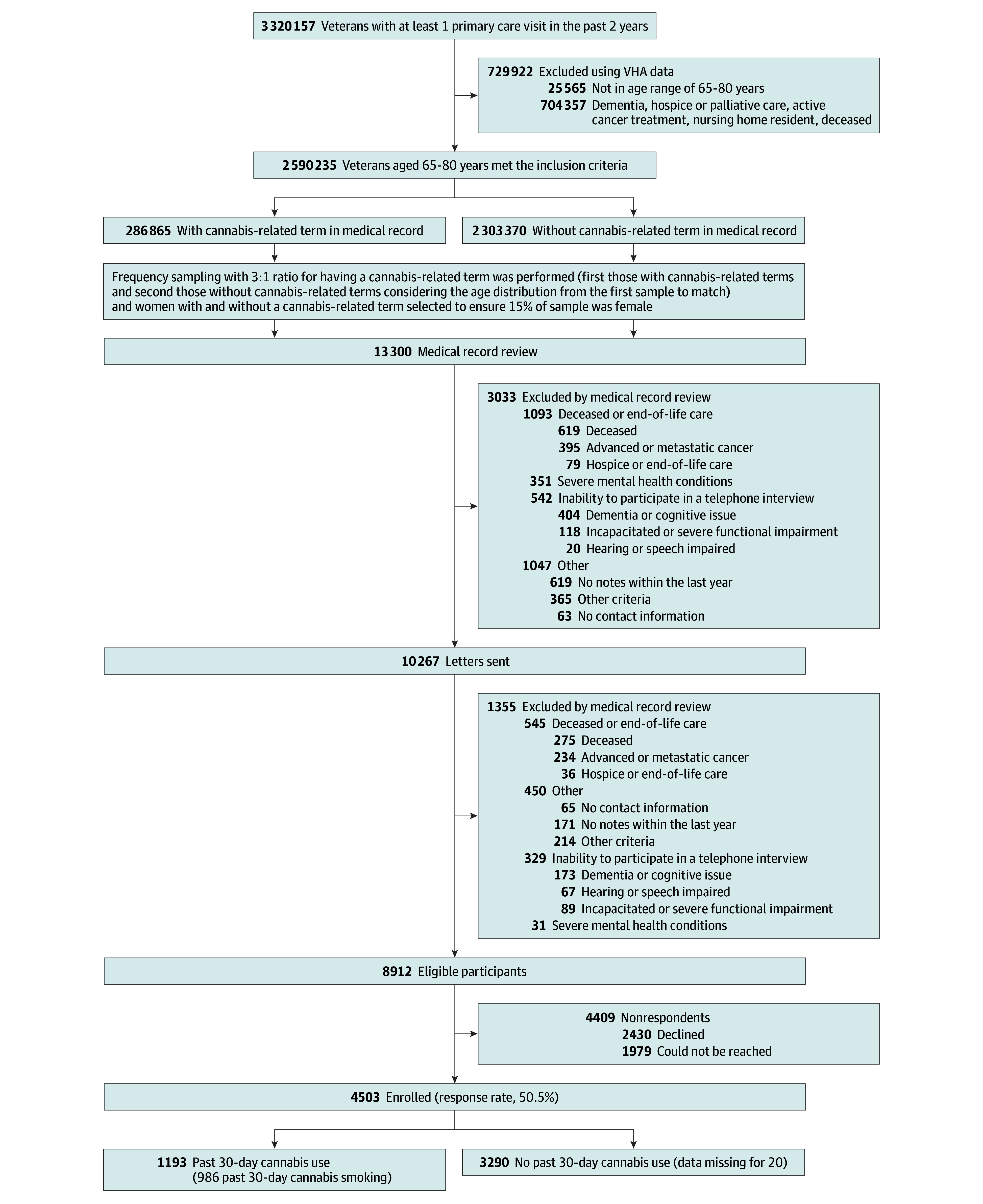
Study Flow Diagram for the Participants VHA indicates Veterans Health Administration.

This study is an analysis of cross-sectional interview and medical record data collected from participants in the VA Cannabis and Aging Cohort at study entry. It includes a national sample of community-dwelling adult VHA patients aged 65 to 84 years with at least 1 primary care visit in the 2 years before October 3, 2019, to ensure that we captured a population who would have baseline data available in the VHA records (n = 2 590 235). Using the Corporate Data Warehouse (CDW), we excluded 729 922 patients (22.0%) who were not in the age range of interest, deceased, or potentially at the end of life (eg, hospice or palliative care) (Figure 1). Participants provided verbal informed consent before the interview. The University of California, San Francisco Institutional Review Board provided a waiver for obtaining written consent. This study was approved by the University of California, San Francisco Institutional Review Board and followed the Strengthening the Reporting of Observational Studies in Epidemiology (STROBE) reporting guideline for cross-sectional studies.

The VA Cannabis and Aging Study selected people with and without cannabis use by identifying adults with medical record documentation of prior cannabis use or nonuse by applying a previously developed text processing algorithm.^55^ We categorized potential participants into 2 groups: those with (n = 286 865) and without (n = 2 303 370) a term denoting cannabis use in their medical record in the past 6 months (eg, *cannabis, marijuana, MJ*). To recruit a mix of those initially identified from both groups (with and without cannabis-related terms), we randomly selected individuals who potentially engaged (3.6% sampled) and did not engage (0.1% sampled) in cannabis use. To ensure sufficient representation, we also oversampled female veterans to ensure that approximately 15% of the sample was female. We sampled participants until we met recruitment goals.

Once participants had been selected, we reviewed 13 300 medical records (eg, health professionals’ summaries) to identify those with exclusion criteria missed by VHA CDW data in the first round (eg, end-of-life care). We excluded 3033 participants (22.8%) in 4 categories using medical record reviews: (1) those deceased or receiving end-of-life care (n = 1093); (2) those with a severe mental health condition (eg, active suicidal ideation documented in a recent visit), for whom a phone call might have exacerbated the underlying mental health condition and who could not participate in an interview (n = 351); (3) those unable to participate in a telephone interview (eg, having a severe cognitive or speech impairment) (n = 542); and (4) other (eg, no contact information, lived outside the US, or incarcerated) (n = 1047). Eligible participants (n = 10 267) were mailed a letter and then telephoned after 2 weeks and invited to participate in a 30- to 45-minute telephone interview (the initial incentive of US $25 was later increased to US $35). At this stage, we identified an additional 1355 patients (13.2%) who met the 4 exclusion criteria before or during interviews. From the sample of eligible individuals (n = 8912), we recruited respondents from February 5, 2020, to August 29, 2023 (Figure 1; eTable 1 in Supplement 1). Study data were collected and managed using REDCap (Research Electronic Data Capture) electronic data capture tools hosted at the VA.^56^

### Assessment of Cannabis Use

The telephone interview asked about forms and frequency of lifetime, last-year, and past 30-day cannabis use and about duration (in years) of lifetime cannabis smoking (eTable 2 in Supplement 1). Respondents reported reasons for lifetime use of medical or recreational cannabis and specified for which health concerns they had ever used cannabis (eg, insomnia or pain) (eTable 3 in Supplement 1). Those who reported past 30-day cannabis use were asked about 11 CUD-related diagnostic criteria related to their experiences in the past 12 months (eTable 2 in Supplement 1) using a checklist derived from the CUD questions included in the *Diagnostic and Statistical Manual of Mental Disorders* (Fifth Edition) (*DSM-5*)^57^: mild CUD, defined as reporting 2 to 3 criteria; moderate CUD, defined as reporting 4 to 5 criteria; and severe CUD, defined as reporting 6 criteria or more.

### Assessments of Baseline Health

#### Sociodemographic Characteristics

Using data from the VHA CDW, we collected data regarding respondents’ age, race and ethnicity, employment status, and state of residence. During the survey interview, respondents provided data on their marital status, the highest level of educational attainment, and whether it was hard to pay for basic needs as a proxy for income. Race and ethnicity were included in the analysis because one of the primary goals of the study was to examine sociodemographic correlates of cannabis use and CUD among older veterans.

#### Health Behaviors

Respondents provided information on current and past tobacco use status, alcohol use, and past 30-day illicit drug use (eg, amphetamines or cocaine) (eTable 2 in Supplement 1). Using interview data on quantity and frequency of past-year alcohol use, we assessed the presence or absence of hazardous drinking with the Alcohol Use Disorders Identification Test–Consumption (AUDIT-C) (cutoff scores of ≥4 for men and ≥3 for women).^58^

#### Mental Health

Respondents self-reported feeling lonely vs not lonely based on a 3-item scale.^59^ We assessed dichotomized variables indicating PTSD (cutoff score >18 based on the 8-item Posttraumatic Stress Disorder Checklist for *DSM-5*^60^), moderate-severe anxiety (>9 based on the 7-item Generalized Anxiety Disorder screener^61^), and clinically significant depressive symptoms (≥10 based on the 8-item Patient Health Questionnaire screener^62^).

#### Physical Health and Aging Relevant Variables

Using electronic health record (EHR) data, we examined the Care Assessment Need score (categorized into 3 groups: <50, 50 to <75, and ≥75), which is a VHA-specific measure where a higher score indicates higher hospitalization and mortality risk,^63,64^ and a dichotomized variable indicating an individual’s independence in ADLs based on the Katz Index of Independence in Activities of Daily Living.^21,65^ Respondents also reported experiencing falls in the past 12 months (yes or no).

### Outcome Variables

We examined 2 main outcome variables. The first was a binary variable of self-reported past 30-day cannabis use, defined as use of inhaled (smoking, vaporizing or “vaping,” or dabbing) or edible cannabis because of higher health risk associated with these routes of administration vs topical use (ie, topicals were coded as no past 30-day use). The second was a binary variable indicating any past 12-month CUD (≥2 of the 11 CUD-related diagnostic criteria) (eTable 2 in Supplement 1) among those with past 30-day cannabis use.^57^ We descriptively reported the prevalence of cannabis use in the last year and in a lifetime (including and excluding topicals) and frequent past 30-day cannabis use (ie, ≥20 days, similar to prior surveys^3^).

### Statistical Analysis

All analyses were conducted in SAS software, version 9.4 (SAS Institute Inc) from February to May 2024 using PROC SURVEY procedures with combined person-level analytic weights (sampling weights × nonresponse weights) to account for the sampling of our cohort from the larger population of VHA patients (our target population) and nonresponse to the patient survey. The denominator for our cohort sampling weights came from the full VHA patient population; sampling weights were computed using age, gender, race, ethnicity, US Census region, and presence of cannabis-related terms in medical notes. These sampling weights were applied to align the sample with the distribution of veterans aged 65 to 80 years who had met study inclusion criteria (n = 2 590 235) (Figure 1). Nonresponse weights were computed using the same factors to adjust for differences between respondents and nonrespondents. The final person-level weights, which were the product of sampling and nonresponse weights, were then normalized to ensure the weighted sample also summed to the total population of veterans aged 65 to 80 years who had met the inclusion criteria (n = 2 590 235).

For descriptive analysis, we reported weighted means with 95% CIs or medians with IQRs for continuous variables; for categorical variables, we reported unweighted numbers and weighted percentages together with 95% CIs for the total sample, by gender, by lifetime or past 30-day cannabis use, and by CUD status. We compared weighted percentages via the Rao-Scott χ^2^ tests by gender, past 30-day cannabis use, and CUD.

Our primary adjusted analysis assessed correlates of past 30-day cannabis use among all respondents and of CUD among those reporting past 30-day use. We used weighted multivariable logistic regression models, simultaneously including sociodemographic (age, gender, race and ethnicity, educational level, employment, economic hardship, marital status, and state of residents) and behavioral (alcohol, tobacco, and illicit drug use) characteristics, self-reported loneliness, and mental health concerns (anxiety, depression, or PTSD) as well as physical function or health-related factors (Care Assessment Need score, ADLs, or experiencing falls). The CUD model also included the frequency of past 30-day cannabis use as an independent variable.

Our secondary adjusted analysis assessed the odds of CUD with the forms of past 30-day cannabis use among those reporting past 30-day cannabis use (model 1: smoking, vaping, dabbing, and use of edibles separately; model 2: any inhaled cannabis use vs edibles only). We used weighted multivariable logistic regression models that controlled for age, gender, frequency of past 30-day cannabis use, and reasons for lifetime cannabis use. We then performed 2 sensitivity analyses. First, we reran models 1 to 2 without accounting for frequency of use to examine whether it was confounding associations between any CUD and forms of use. Second, we reran models 1 to 2 of the secondary adjusted analysis while defining CUD as reporting 4 or more CUD-related criteria (ie, any moderate or severe CUD).

Our collinearity diagnostics revealed no substantial collinearity among the included variables in the models (variance inflation factors were <1.82). In all analyses, we used listwise deletion of missing data; the combined proportion of missing values for the response and independent variables in each analysis was less than 2.5%. Two-tailed *P* < .05 was considered statistically significant.

## Results

A total of 4503 respondents participated in the study (response rate, 50.5%; 51.3% among those with the *cannabis* term in the medical records and 48.2% among those without the *cannabis* term) (Figure 1). The study participants were not substantially different from the nonrespondents (eTable 1 in Supplement 1), and there were no significant differences by age, race, and ethnicity between respondents and nonrespondents when stratified by a *cannabis* term noted in the medical records. Among the 4503 respondents, the weighted mean age was 73.3 years (95% CI, 73.0-73.5 years). Of the respondents, 4.2% (95% CI, 3.1%-5.4%) were Hispanic, 13.3% (95% CI, 11.6%-15.0%) were non-Hispanic Black or African American, 78.4% (76.2%-80.6%) were non-Hispanic White, and 4.0% (95% CI, 2.9%-5.1%) were non-Hispanic other race (Asian, American Indian or Alaska Native, Native Hawaiian or Other Pacific Islander, multiple races, or unknown or not reported). Most were men (85.4%; 95% CI, 83.6%-87.2%), retired (73.3%; 95% CI, 70.9%-75.8%), and married (62.6%; 95% CI, 59.9%-65.3%). Most had a high school degree or received some college education (66.7%; 95% CI, 64.0%-69.4%) and reported no hardship in paying for basic needs (81.5%; 95% CI, 79.4%-83.6%) (Table 1). The survey included residents of 50 states as well as Washington, DC, and Puerto Rico.

**Table 1.** Characteristics of the Sample of US Veterans, 2020-2023^a^

Characteristic	No. (weighted %) [95% CI]			*P* value^b^
	Total (N = 4503)	Men (3689 [85.4%])	Women (814 [14.6%])	
Age group, y				
65-70	1695 (23.9) [21.9-25.8]	1307 (20.8) [18.8-22.8]	388 (42.1) [35.9-48.2]	<.001
71-75	2094 (49.2) [46.3-52.0]	1801 (51.6) [48.5-54.7]	293 (34.7) [28.6-40.8]	
76-84	714 (27.0) [24-29.9]	581 (27.6) [24.4-30.9]	133 (23.2) [16.4-29.9]	
Race and ethnicity^c^				
Black or African American, non-Hispanic	844 (13.3) [11.6-15.0]	663 (11.8) [10.1-13.6]	181 (22.2) [16.9-27.5]	<.001
Hispanic	219 (4.2) [3.1-5.4]	196 (4.7) [3.4-6.0]	23 (1.6) [0.1-3.1]	
White, non-Hispanic	3253 (78.4) [76.2-80.6]	2688 (79.6) [77.3-81.9]	565 (71.4) [65.6-77.2]	
Other race, non-Hispanic	187 (4.0) [2.9-5.1]	142 (3.9) [2.7-5.1]	45 (4.8) [2.2-7.4]	
Marital status				
Married, partner, or engaged	2326 (62.6) [59.9-65.3]	2105 (67.6) [64.8-70.4]	221 (33.2) [27-39.3]	<.001
Other (eg, single, divorced)	2177 (37.4) [34.7-40.1]	1584 (32.4) [29.6-35.2]	593 (66.8) [60.7-73]	
Educational level				
Bachelor’s degree and beyond	1229 (30.7) [28.1-33.3]	881 (28.6) [25.8-31.5]	348 (42.8) [36.4-49.2]	<.001
High school graduate, some college	3100 (66.7) [64.0-69.4]	2636 (68.4) [65.4-71.3]	464 (57.1) [50.7-63.5]	
Less than high school graduate	174 (2.6) [1.7-3.4]	172 (3.0) [2.0-4.0]	2 (0.1) [0.0-0.1]	
Employment (data missing for 2 [0.01%])				
Employed	438 (11.5) [9.7-13.2]	368 (12.0) [10.1-14.0]	70 (8.2) [4.9-11.4]	.11
Retired	2971 (73.3) [70.9-75.8]	2437 (73.3) [70.6-76.0]	534 (73.6) [68.1-79.1]	
Other	1092 (15.2) [13.3-17.1]	882 (14.7) [12.6-16.7]	210 (18.2) [13.4-23.0]	
Hard to pay for basic needs (data missing for 9 [0.2%])	1167 (18.3) [16.2-20.4]	906 (17.0) [14.8-19.2]	261 (26.1) [20.4-31.8]	.001
Not very hard	3327 (81.5) [79.4-83.6]	2776 (82.9) [80.7-85.1]	551 (73.2) [67.4-79.0]	
Has ever used cannabis (data missing for 14 [0.4%])^d^	3420 (58.2) [55.3-61.0]	2827 (58.0) [54.9-61.1]	593 (59.3) [52.7-66.0]	.77
Past 30-d cannabis use^e^ (data missing for 20 [0.4%])				
Current (past 30 d)	1193 (10.3) [8.9-11.7]	1018 (10.2) [8.6-11.7]	175 (11.1) [7.6-14.5]	.90
No current (more than past 30 d)	2221 (47.9) [45.1-50.7]	1803 (47.8) [44.7-50.9]	418 (48.3) [41.8-54.8]	
Never	1069 (41.5) [38.6-44.3]	849 (41.6) [38.5-44.7]	220 (40.6) [34.0-47.3]	
Past 30-d forms of cannabis use				
Smoking (data missing for 18 [0.4%])	986 (7.4) [6.3-8.6]	869 (7.7) [6.5-8.9]	117 (5.9) [3.5-8.3]	.23
Vaping (data missing for 20 [0.5%])	170 (1.2) [0.8-1.6]	141 (1.1) [0.7-1.6]	29 (1.4) [0.4-2.5]	.60
Dabbing (data missing for 23 [0.6%])	32 (0.2) [0.1-0.3]	28 (0.2) [0.1-0.3]	4 (0.1) [0.0-0.2]	.33
Edibles (data missing for 26 [0.5%])	339 (3.8) [2.8-4.7]	257 (3.2) [2.2-4.2]	82 (7.4) [4.3-10.5]	.001
Frequent past 30-d cannabis use (≥20 d; data missing for 21 [0.4%])	753 (5.4) [4.4-6.4]	650 (5.5) [4.4-6.6]	103 (5.0) [2.9-7.0]	
Nonfrequent past 30-d use	439 (4.9) [3.9-5.9]	367 (4.7) [3.6-5.8]	72 (6.1) [3.2-9.0]	.53
No past 30-d use	3290 (89.3) [87.9-90.8]	2652 (89.4) [87.8-91]	638 (88.9) [85.4-92.4]	
Any past 12-mo cannabis use disorder (≥2 criteria)^f^	546 (3.7) [3.0-4.4]	490 (3.9) [3.1-4.7]	56 (2.7) [1.1-4.4]	.23
Past 12-mo cannabis use disorder criteria				
0	433 (4.8) [3.7-5.9]	343 (4.4) [3.3-5.6]	90 (6.9) [4.0-9.9]	.25
1	211 (1.7) [1.2-2.3]	182 (1.8) [1.1-2.5]	29 (1.4) [0.4-2.4]	
2-3 (Mild)	336 (2.4) [1.8-2.9]	306 (2.5) [1.8-3.1]	30 (1.7) [0.3-3.2]	
4-5 (Moderate)	144 (1.1) [0.7-1.5]	127 (1.2) [0.7-1.6]	17 (0.8) [0.0-1.5]	
≥6 (Severe)	66 (0.3) [0.2-0.3]	57 (0.3) [0.2-0.3]	9 (0.2) [0.1-0.4]	
Not applicable	3313 (89.7) [88.3-91.1]	2674 (89.9) [88.3-91.4]	639 (88.9) [85.5-92.4]	
Medical cannabis legalization status (vs not legal)	3152 (66.1) [63.4-68.7]	2620 (66.6) [63.7-69.5]	532 (63.1) [56.8-69.4]	.32
Recreational and medical cannabis legalization status (vs not legal)	1694 (29.9) [27.3-32.4]	1374 (29.3) [26.5-32.1]	320 (33.4) [27.4-39.5]	.21
State cannabis legalization status				
Nonlegal	1351 (33.9) [31.3-36.6]	1069 (33.4) [30.5-36.3]	282 (36.9) [30.6-43.2]	.10
Medical only	1458 (36.2) [33.5-38.9]	1246 (37.3) [34.3-40.3]	212 (29.7) [23.6-35.8]	
Recreational and medical	1694 (29.9) [27.3-32.4]	1374 (29.3) [26.5-32.1]	320 (33.4) [27.4-39.5]	
Any past 30-d tobacco use (any tobacco smoking or e-cigarette use; data missing for 5 [0.2%])				
Current (>16 d in the past month)	800 (11.7) [10.1-13.4]	666 (11.6) [9.8-13.4]	134 (12.6) [8.3-16.8]	<.001
No current use	2521 (56.8) [54.0-59.5]	2173 (60.0) [56.9-63]	348 (38.0) [31.7-44.2]	
Never	1177 (31.3) [28.7-34.0]	846 (28.3) [25.4-31.2]	331 (49.1) [42.6-55.6]	
AUDIT-C (data missing for 9 [0.1%])				
Negative	3365 (75.5) [73.1-77.9]	2707 (75.1) [72.4-77.7]	658 (78.1) [72.9-83.3]	.34
Positive	1129 (24.4) [22.0-26.8]	973 (24.8) [22.1-27.4]	156 (21.9) [16.7-27.1]	
Past 30-d illicit drug use (data missing for 25 [0.3%])^g^	57 (0.3) [0.1-0.4]	56 (0.3) [0.2-0.4]	1 (0.0) [0.0-0.1]	.003
≥10 Symptoms based on GAD-7 (data missing for 3 [0.1%])	814 (11.7) [10.0-13.4]	643 (10.5) [8.7-12.2]	171 (19.1) [13.8-24.3]	<.001
≥10 Depressive symptoms based on PHQ-8 (data missing for 8 [0.1%])	986 (13.1) [11.3-14.9]	772 (12.0) [10.1-13.9]	214 (19.5) [14.4-24.7]	.002
≥19 Symptoms based on PCL-8 inventory (data missing for 7 [0.1%])	449 (6.0) [4.8-7.3]	344 (5.3) [4.0-6.6]	105 (10.3) [6.3-14.3]	.005
Loneliness (data missing for 3 [0.01%])				
Not lonely	3285 (81.7) [79.6-83.8]	2759 (83.1) [80.9-85.3]	526 (73.4) [67.9-78.9]	<.001
Lonely	1215 (18.3) [16.2-20.3]	927 (16.9) [14.7-19.1]	288 (26.6) [21.1-32.1]	
Getting social support in COVID-19 (data missing for 14 [0.2%])				
Extremely or very difficult	167 (2.5) [1.6-3.3]	134 (2.3) [1.4-3.3]	33 (3.2) [1.1-5.2]	.44
Other	4322 (97.3) [96.4-98.2]	3541 (97.4) [96.4-98.4]	781 (96.8) [94.8-98.9]	
CAN score (data missing for 9 [0.5%])				
<50	1274 (27.5) [25.1-29.8]	892 (23.9) [21.4-26.3]	382 (48.6) [42.1-55.1]	<.001
50 to <75	1953 (43.1) [40.3-45.8]	1631 (43.5) [40.4-46.5]	322 (40.6) [34.0-47.2]	
≥75	1267 (29.0) [26.3-31.8]	1161 (32.3) [29.2-35.4]	106 (9.7) [5.6-13.8]	
≥1 Deficit in activities of daily life support (data missing for 16 [0.4%])	1663 (32.3) [29.7-35.0]	1176 (28.6) [25.7-31.4]	487 (54.6) [48.1-61.0]	<.001
Falls in the past 12 mo (data missing for 3 [0.01%])	1740 (34.4) [31.7-37.1]	1338 (32.7) [29.7-35.6]	402 (44.5) [38.1-51.0]	<.001

Abbreviations: AUDIT-C, Alcohol Use Disorders Identification Test–Consumption; CAN, Care Assessment Need; GAD-7, 7-item Generalized Anxiety Disorder; PCL-8, 8-item Posttraumatic Stress Disorder Checklist for the *Diagnostic and Statistical Manual of Mental Disorders*; PHQ-8, 8-item Patient Health Questionnaire.

^a^ Data are unweighted numbers and weighted percentages together with the 95% CIs for the total sample (unless otherwise specified) and by gender.

^b^ *P* values shown are for comparisons that included 3 groups: (1) those who reported past 30-day cannabis use with cannabis use disorder (CUD), those who reported past 30-day cannabis use without CUD, and (3) not applicable (no use or missing). *P* values shown are for *F* statistics from Rao-Scott χ^2^ tests for categorical variables while excluding missing or noneligible respondents.

^c^ The White, non-Hispanic category also included respondents who identified as White of unknown ethnicity (n = 85 [1.2%]); the Black or African American, non-Hispanic category also included respondents who identified as Black or African American of unknown ethnicity (n = 13 [0.3%]). The other race, non-Hispanic category included respondents of another race (31 [0.8%] Asian, 50 [1.0%] American Indian or Alaska Native, and 43 [0.6%] Native Hawaiian or Other Pacific Islander), of multiple races (n = 1 [0.004%]), or of unknown or unreported race or ethnicity (62 [1.6%]).

^d^ Affirmative responses to the question, “Have you ever used marijuana in any form?” regardless of the form of use.

^e^ Smoking, vaping, dabbing, or use of edibles in the past 30 days.

^f^ Cannabis use disorder was defined as reporting any 2 or more of the following 11 criteria among those who reported past 30-day use: tolerance; withdrawal or high use; taken in larger amounts or during a longer period than intended; a great deal of time getting, using, or recovering from use; important activities given up; recurrent psychological or physical problems; hazardous use; recurrent social problems; failure to fulfill obligations; craving; and/or persistent desire to cut down.

^g^ Includes cocaine, heroin, amphetamines, or other illicit drugs.

### Lifetime Cannabis Use

More than half of the respondents (57.4%; 95% CI, 54.6%-60.3%) reported lifetime cannabis use in the form of smoking, vaping, dabbing, or edibles, and 14.1% (95% CI, 12.5%-15.8%) reported last-year use (eFigure 1 in Supplement 1). Smoking cannabis was the most common form (97.2%; 95% CI, 96.0%-98.4%), followed by the use of edibles (34.3%; 95% CI, 31.2%-37.4%) (eFigure 2 in Supplement 1).

Among those with available data on forms of lifetime cannabis use including topicals (n = 3409), 28.9% (95% CI, 26.0%-31.8%) reported any medical use: 16.7% (95% CI, 14.5%-18.8%) used cannabis for both medical and recreational purposes, and 12.3% (95% CI, 10.1%-14.5%) used medicinal cannabis only (eFigure 3 in Supplement 1). The most common reasons for any medical use were management of pain (any: 56.4%; 95% CI, 50.9%-61.9%; general or vascular: 33.4%; 95% CI, 28.1%-38.6%; muscle or skeletal: 24.8%; 95% CI, 19.9%-29.7%), sleep difficulties (16.0%; 95% CI, 11.9%-20.0%), arthritis (11.3%; 95% CI, 8.0%-14.6%), and mood or mental health concerns (any: 18.4%; 95% CI, 14.7%-22.1%; anxiety: 9.9%; 95% CI, 6.9%-13.0%; PTSD: 6.8%; 95% CI, 4.6%-9.0%; depression: 4.5%; 95% CI, 3.1%-5.9%) (eFigure 4 in Supplement 1).

### Past 30-Day Use

**Figure 2.**
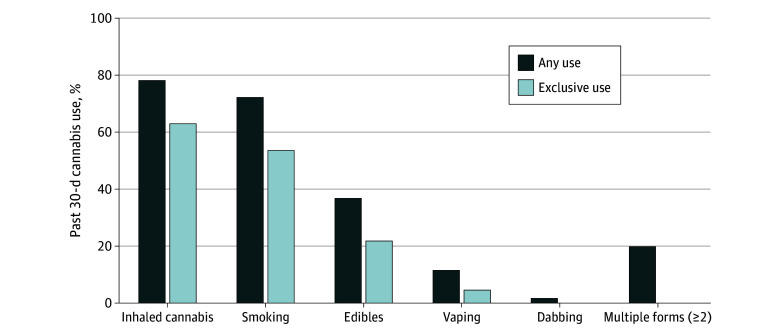
Forms of Past 30-Day Cannabis Use Among US Veterans 65 Years and Older, 2020-2023 Results shown are weighted percentages for any and exclusive use of cannabis by modes of use (smoking, vaping, dabbing, or edibles) among those who used cannabis in the past 30 days (n = 1193 [10.3% of the total sample of 4503]). Any use indicates those who reported any cannabis use in the past 30 days by the forms shown (eg, any use of inhaled cannabis includes 78.3% of those who reported any smoking, vaping, and/or dabbing regardless of the use of edibles). Exclusive use indicates those who reported cannabis use exclusively by the forms shown (eg, exclusive use of inhaled cannabis includes 63.1% of those who reported any inhaled use [smoking, vaping, or dabbing] and no edibles). In contrast, 21.7% of respondents with past 30-day cannabis use reported the use of edible cannabis only. Multiple forms includes reporting any 2 or more forms of use (smoking, vaping, dabbing, and/or edibles).

More than 1 in 10 respondents (10.3%; 95% CI, 8.9%-11.7%) reported past 30-day cannabis use (Table 1). More women than men used edibles in the past month (7.4% [95% CI, 4.3%-10.5%] vs 3.2% [95% CI, 2.2%-4.2%]; *P* = .001), whereas other forms of use did not differ by gender (Table 1). Among those with past 30-day cannabis use (n = 1193), smoking (72.4%; 95% CI, 65.4%-79.3%; median [IQR] years of daily cannabis smoking, 19.1 [1.3-39.4]) and use of edibles (36.9%; 95% CI, 29.8%-43.9%) were the most common, and 19.9% (95% CI, 14.9%-24.9%) reported 2 or more forms of use (Figure 2). Many of those who reported edible cannabis use also inhaled cannabis (41.2%; 95% CI, 29.4%-52.9%). More than half of those with past 30-day use (52.4%; 95% CI, 45.4%-59.4%) used cannabis frequently.

In the weighted multivariable logistic regression analysis, past 30-day cannabis use (vs no past 30-day use) was more likely reported among younger respondents (aged 65-70 vs 76-84 years: adjusted odds ratio [AOR], 2.86; 95% CI, 1.59-5.17; aged 71-75 vs 76-84 years: AOR, 2.04; 95% CI, 1.13-3.69); participants who were not (vs were) married or in a relationship (AOR, 1.85; 95% CI, 1.30-2.64); those who were not retired or employed vs retired (AOR, 1.55; 95% CI, 1.07-2.26); those who experienced economic hardships (vs those who did not) (AOR, 1.61; 95% CI, 1.05-2.45); those who reported current tobacco use (AOR, 2.78; 95% CI, 1.66-4.64) and past tobacco use (AOR, 1.92; 95% CI, 1.19-3.10) vs no lifetime use; those with any illicit drug use (vs none) in the past month (AOR, 2.72; 95% CI, 1.14-6.49); those who had a positive (vs negative) AUDIT-C score (AOR, 1.47; 95% CI, 1.04-2.06); and those who resided in states with recreational (vs nonlegal) cannabis status (AOR, 2.53; 95% CI, 1.71-3.75) (Table 2).

**Table 2.** Correlates of Past 30-Day Cannabis Use for Respondents With Available Data on Cannabis Smoking, Vaping, Dabbing, and Use of Edibles in 4483 US Veterans, 2020-2023^a^

Characteristic	No. (weighted %) [95% CI]		AOR (95% CI)^c^	*P* value^c^
	Past 30-d use (n = 1193)^b^	No past 30-d use (n = 3290)		
Age group, y				
65-70	558 (36.0) [30.0-41.9]	1126 (22.5) [20.4-24.6]	2.86 (1.59-5.17)	<.001
71-75	521 (49.2) [42.3-56.2]	1565 (49.2) [46.1-52.2]	2.04 (1.13-3.69)	.02
76-84	114 (14.8) [8.1-21.6]	599 (28.4) [25.2-31.5]	1 [Reference]	NA
Gender				
Men	1018 (84.3) [79.6-89.1]	2652 (85.5) [83.6-87.5]	1.03 (0.62-1.71)	.90
Women	175 (15.7) [10.9-20.4]	638 (14.5) [12.5-16.4]	1 [Reference]	NA
Race and ethnicity				
Black or African American, non-Hispanic	259 (18.0) [13.3-22.7]	582 (12.8) [11.0-14.7]	1.17 (0.79-1.75)	.43
Hispanic	53 (4.8) [1.9-7.8]	166 (4.2) [3.0-5.4]	0.98 (0.49-1.96)	.95
White, non-Hispanic	835 (73.6) [68.0-79.3]	2402 (78.9) [76.5-81.2]	1 [Reference]	NA
Other race, non-Hispanic^d^	46 (3.6) [1.3-5.9]	140 (4.1) [2.9-5.3]	0.82 (0.35-1.93)	.66
Marital status				
Other	669 (55.4) [48.4-62.3]	1500 (35.4) [32.6-38.3]	1.85 (1.30-2.64)	<.001
Married, partner, or engaged	524 (44.6) [37.7-51.6]	1790 (64.6) [61.7-67.4]	1 [Reference]	NA
Educational level				
Less than high school graduate	53 (3.0) [1.4-4.5]	121 (2.5) [1.6-3.5]	0.77 (0.36-1.64)	.50
High school graduate or some college	858 (69.8) [63.3-76.2]	2230 (66.5) [63.6-69.4]	0.85 (0.56-1.28)	.43
Bachelor’s degree and beyond	282 (27.3) [20.9-33.7]	939 (30.9) [28.1-33.8]	1 [Reference]	NA
Employment (n = 4480)				
Employed	91 (7.6) [4.0-11.1]	343 (11.8) [9.9-13.7]	0.56 (0.30-1.03)	.06
Other	332 (25.2) [19.4-31.1]	755 (14.1) [12.0-16.1]	1.55 (1.07-2.26)	.02
Retired	770 (67.2) [60.8-73.6]	2190 (74.1) [71.5-76.7]	1 [Reference]	NA
Hard to pay for basic needs (n = 4474)^e^	347 (28.8) [22.3-35.3]	811 (17.2) [15.0-19.4]	1.61 (1.05-2.45)	.03
Not very hard	844 (71.1) [64.6-77.6]	2472 (82.7) [80.5-84.9]	1 [Reference]	NA
State cannabis legalization status				
Nonlegal	299 (24.1) [18.6-29.6]	1046 (35.1) [32.2-38.0]	1 [Reference]	NA
Medical only	390 (33.5) [26.8-40.1]	1062 (36.6) [33.6-39.6]	1.43 (0.94-2.16)	.10
Recreational and medical	504 (42.4) [35.5-49.4]	1182 (28.3) [25.6-31.0]	2.53 (1.71-3.75)	<.001
Any past 30-d tobacco use (n = 4479)^f^				
Current (>16 d in the past month)	347 (21.8) [16.9-26.7]	451 (10.6) [8.9-12.4]	2.78 (1.66-4.64)	<.001
No current use	674 (59.5) [52.7-66.2]	1833 (56.4) [53.4-59.4]	1.92 (1.19-3.10)	.007
Never	172 (18.7) [12.8-24.6]	1002 (32.8) [29.9-35.7]	1 [Reference]	NA
AUDIT-C (n = 4476)				
Positive	415 (32.3) [26.1-38.6]	706 (23.3) [20.8-25.9]	1.47 (1.04-2.06)	.03
Negative	776 (67.6) [61.4-73.9]	2579 (76.5) [73.9-79.1]	1 [Reference]	NA
Past 30-d illicit drug use (n = 4463)	32 (1.2) [0.7-1.6]	25 (0.2) [0.0-0.3]	2.72 (1.14-6.49)	.02
≥10 Symptoms based on the GAD-7 (n = 4480)	187 (12.3) [7.9-16.7]	625 (11.7) [9.8-13.5]	0.73 (0.41-1.29)	.28
≥10 Depressive symptoms based on the PHQ-8 (n = 4475)	251 (17.5) [12.4-22.6]	730 (12.6) [10.7-14.5]	1.44 (0.86-2.42)	.17
≥19 Symptoms based on PCL-8 inventory (n = 4476)	99 (5.6) [3.2-8.0]	348 (6.1) [4.7-7.5]	0.69 (0.36-1.31)	.26
Loneliness (n = 4480)				
Not lonely	849 (77.2) [71.8-82.7]	2423 (82.2) [80.0-84.4]	1 [Reference]	NA
Lonely	342 (22.7) [17.2-28.2]	866 (17.8) [15.6-20.0]	0.96 (0.64-1.43)	.83
CAN score (n = 4474)				
<50	308 (27.7) [21.8-33.7]	962 (27.5) [25.0-30.1]	1 [Reference]	NA
50 to <75	539 (42.7) [35.9-49.5]	1405 (43.1) [40.1-46.1]	1.12 (0.78-1.61)	.54
≥75	343 (29.4) [22.6-36.2]	917 (28.9) [25.9-31.9]	1.30 (0.84-2.02)	.23
≥1 Deficit in activities of daily life support (n = 4467)	409 (32.3) [25.9-38.8]	1248 (32.4) [29.5-35.3]	0.96 (0.65-1.44)	.86
Falls in the past 12 mo (n = 4480)	420 (29.3) [23.4-35.1]	1314 (34.9) [32.0-37.8]	0.71 (0.50-1.03)	.07

Abbreviations: AOR, adjusted odds ratio; AUDIT-C, Alcohol Use Disorders Identification Test–Consumption; CAN, Care Assessment Need; GAD-7, 7-item Generalized Anxiety Disorder; NA, not applicable; PCL-8, 8-item Posttraumatic Stress Disorder Checklist for the *Diagnostic and Statistical Manual of Mental Disorders*; PHQ-8, 8-item Patient Health Questionnaire.

^a^ Data are unweighted numbers and weighted percentages together with the 95% CIs by past 30-day cannabis use for respondents with available data on past 30-day cannabis use (n = 4483; data missing for 20 [0.4%]).

^b^ Smoking, vaping, dabbing, or use of edibles in the past 30 days.

^c^ Results obtained from adjusted logistic regression with all listed variables entered simultaneously; past 30-day use is the outcome variable: 4403 observations used (data missing for 100 [2.2%] of the total sample): 1166 reporting past 30-day cannabis use vs 3237 reporting no past 30-day use.

^d^ The other race, non-Hispanic category included respondents of another race (31 [0.8%] Asian, 50 [1.0%] American Indian or Alaska Native, and 42 [0.6%] Native Hawaiian or Other Pacific Islander), of multiple races (1 [0.004%]), or of unknown or not reported race or ethnicity (62 [1.6%]).

^e^ Reported very hard, hard, or somewhat hard to pay for basic needs or reported being homeless, living in a shelter, a mobile home, or in a single room in a building.

^f^ Smoking cigarettes, cigarillos, cigars, or pipes or using e-cigarettes for more than 16 days in the past month.

### Cannabis Use Disorder

Of 1190 respondents with past 30-day cannabis use and available data on CUD diagnostic criteria, 36.3% (95% CI, 30.1%-42.6%) met our past 12-month CUD definition (or 3.7% [95% CI, 3.0%-4.4%] in the total sample) (Table 1; eFigure 1 in Supplement 1), including 22.9% (95% CI, 17.7%-28.1%) with mild, 10.9% (95% CI, 7.0%-14.8%) with moderate, and 2.5% (95% CI, 1.8%-3.2%) with severe CUD (eFigure 5 in Supplement 1). In the weighted multivariable logistic regression analysis, men, respondents younger than 76 years, those who experienced anxiety, those who reported frequent cannabis use or any illicit drug use in the past month, and those who reported 1 or more deficits in ADLs were more likely to have any CUD (eTable 4 in Supplement 1). Lifetime cannabis use for exclusively medical reasons compared with any recreational use (recreational only or combined with medical use) was associated with lower odds of any CUD (≥2 criteria). Frequent past 30-day cannabis use was the highest among those who had ever used cannabis for both medical and recreational purposes (61.0%; 95% CI, 52.4%-69.6%), followed by those who used medical cannabis only (53.3%; 95% CI, 38.1%-68.6%), and was the lowest among respondents who only used recreational cannabis (30.4%; 95% CI, 15.9%-44.9%) (eFigure 6 in Supplement 1).

### Secondary Analysis: Associations of CUD With Forms and Frequency of Cannabis Use

In the weighted multivariable logistic regression analysis, past 30-day use of any inhaled cannabis vs edibles only was associated with higher odds of any CUD (AOR, 3.56; 95% CI, 1.12-11.26) (Table 3) and vice versa: use of edibles only vs any inhaled cannabis was associated with lower odds of any CUD (model 3) (see eTable 5 in Supplement 1 for reciprocal results). Most respondents who used any inhaled cannabis reported daily or near-daily use (61.0%; 95% CI, 53.9%-68.1%), whereas those who exclusively consumed edibles were less likely to use cannabis frequently (21.5%; 95% CI, 7.3%-35.7%). Frequent past 30-day cannabis use was independently associated with higher odds of any CUD in all models (Table 3).

**Table 3.** Association of Forms of Use With Any Past 12-Month CUD Among 1193 US Veterans Who Reported Any Past 30-Day Cannabis Use Adjusted by Age and Gender, 2020-2023^a^

Characteristic	No. (weighted %) [95% CI]	CUD, No. (%) [95% CI]	Model 1 (CUD vs no CUD)^b^		Model 2 (CUD vs no CUD)^c^	
			AOR (95% CI)^d^	*P* value	AOR (95% CI)^d^	*P* value
Use						
Any smoking	986 (72.4) [65.4-79.3]	494 (43.0) [35.8-50.2]	2.08 (0.87-4.99)	.10	NA	NA
Any vaping	170 (11.5) [7.7-15.3]	83 (43.2) [26.4-59.9]	1.26 (0.47-3.40)	.65		
Any dabbing	32 (1.7) [0.6-2.8]	25 (56.7) [21.2-92.2]	1.10 (0.18-6.79)	.91		
Any edibles	339 (36.9) [29.8-43.9]	132 (27.6) [17.4-37.7]	1.05 (0.53-2.10)	.89		
Frequent past 30-d use^e^	753 (52.4) [45.4-59.4]	392 (44.9) [36.2-53.7]	2.14 (1.24-3.71)	.007	2.02 (1.16-3.51)	.01
Form of use						
Edibles only	135 (21.7) [14.9-28.5]	22 (10.2) [0.62-19.8]	NA	NA	1 [Reference]	NA
Any inhaled use	1058 (78.3) [71.5-85.1]	524 (43.5) [36.5-50.5]	NA	NA	3.56 (1.12-11.26)	.03
Age group, y						
65-70	558 (36.0) [30.0-41.9]	263 (43.6) [35.4-51.8]	5.40 (2.11-13.82)	<.001	5.49 (2.15-14.02)	<.001
71-75	521 (49.2) [42.2-56.2]	241 (38.4) [28.8-48.0]	4.80 (1.80-12.75)	.002	4.96 (1.89-13.03)	.001
76-84	114 (14.8) [8.1-21.6]	42 (11.5) [4.8-18.2]	1.0 [Reference]	NA	1 [Reference]	NA
Gender						
Men	1018 (84.3) [79.6-89.1]	490 (38.4) [31.5-45.4]	1.86 (0.81-4.24)	.14	1.63 (0.71-3.72)	.25
Women	175 (15.7) [10.9-20.4]	56 (24.7) [11.6-37.9]	1.0 [Reference]	NA	1 [Reference]	NA
Reasons for lifetime cannabis use						
Medical only	278 (26.5) [19.9-33.2]	80 (18.3) [9.3-27.3]	0.35 (0.17-0.71)	.004	0.36 (0.18-0.73)	.005
Recreational only or both	912 (73.4) [66.7-80.0]	465 (42.8) [35.4-50.2]	1.0 [Reference]	NA	1 [Reference]	NA

Abbreviations: AOR, adjusted odds ratio; CUD, cannabis use disorder; NA, not applicable.

^a^ Data are unweighted numbers, weighted percentages, and 95% CIs for forms of use (not mutually exclusive) and covariates among those who reported past 30-day cannabis use (n = 1193) and row percentages together with the 95% CIs (for CUD).

^b^ Model 1 variables were reporting smoking, vaping, dabbing, or use of edibles while adjusting for age, gender, frequency of past 30-day cannabis use, and reasons for lifetime cannabis use (n = 1182: 545 with CUD vs 637 without CUD).

^c^ Model 2 variables were a 2-level variable indicating forms of cannabis use (any inhaled cannabis use vs edibles only) while adjusting for age, gender, frequency of past 30-day cannabis use, and reasons for lifetime cannabis use (n = 1189: 545 with CUD vs 644 without CUD).

^d^ The AORs for forms of use are shown in associations with the outcome variable of CUD, which is defined as reporting any 2 of the following 11 criteria: tolerance; withdrawal; cannabis taken in larger amounts or during a longer period than intended; a great deal of time getting, using, or recovering from use; important activities given up; recurrent psychological or physical problems; hazardous use; recurrent social problems; failure to fulfill obligations; craving; and/or persistent desire to cut down.

^e^ Smoking, vaping, dabbing, or using edibles 20 or more days in the past 30 days.

Sensitivity analyses revealed that before adjusting for frequency of use, cannabis smoking was associated with higher odds of any CUD (model 1) (eTable 5 in Supplement 1), whereas the association was nonsignificant after controlling for frequent use (model 1) (Table 3). The finding of lower odds of any CUD with exclusive use of edibles (reciprocally, higher odds of any CUD with any inhaled cannabis) were consistently significant before and after adjustments for frequency of use (models 2-3) (eTable 5 in Supplement 1).

Sensitivity analyses examining associations of forms of use with moderate to severe CUD (≥4 criteria: 13.4%; 95% CI, 9.4%-17.4%) among those with past 30-day cannabis use confirmed our primary findings indicating that inhaled (vs edibles only) cannabis use, especially smoking, was associated with moderate to severe CUD regardless of frequency of use (models 4-5) (eTable 5 in Supplement 1). Unlike our primary models with the overall CUD outcome (≥2 criteria), exclusive medical cannabis use compared with recreational use was no longer significantly associated with lower odds for moderate to severe CUD (models 4-5) (eTable 5 in Supplement 1).

## Discussion

In this cross-sectional study of a nationally representative sample of older veterans 65 years and older who received treatment in the VHA, more than 1 in 10 reported past 30-day cannabis use, suggesting almost 2 times higher prevalence of cannabis use compared with their counterparts 65 years or older in the general population (5.2% in 2022^9^). Cannabis consumption on a daily or near-daily basis among those currently using cannabis was common, and more than one-third of those with past 30-day use had at least mild CUD. Prevalence of past 30-day cannabis use was close to that of past 30-day tobacco use, supporting previous studies showing that increasing cannabis use among US adults is approaching tobacco use rates.^1,15,66,67,68^ Our findings highlight the importance of screening older veterans for frequent and disordered cannabis use and informing older veterans about the risks of developing CUD.

CUD was more prevalent among respondents of younger age (65-75 vs ≥76 years) and those who used illicit drugs, frequently used cannabis, or had anxiety or 1 or more deficits in ADLs. In line with prior research,^41^ respondents reporting lifetime cannabis use exclusively for medical reasons vs recreationally were less likely to report any CUD. However, the odds for moderate to severe CUD did not differ significantly among those who used cannabis recreationally vs for medical reasons only. A novel finding of our secondary analysis was that veterans who used inhaled cannabis had higher adjusted odds of CUD compared with those who used edibles only, independently of frequency of use. In contrast, those who exclusively used edibles were less likely to report CUD. Previous studies have shown that higher potency of cannabis products may increase the risk of CUD.^69,70^ Inhaled modes of cannabis administration, especially vaping and dabbing concentrates, can deliver high levels of tetrahydrocannabinol (THC), which may explain our finding that inhaled forms of cannabis are associated with higher odds of CUD.^71,72,73^

It is concerning that the weighted percentage of CUD among veterans 65 years or older engaging in past 30-day cannabis use (36.3%) was at the higher end of the CUD prevalence reported among people who use cannabis in the general US population.^54,74,75^ Besides differences in the nature of our study being based on VHA patients vs the general population in prior research,^54,74,75^ the comparisons with past observational studies may be complicated by variations in the time interval of cannabis use chosen (ie, lifetime, past-year, or past-month use) and discrepancies in the CUD definition, which was based on the *DSM-5*^57^ in our survey compared with *Diagnostic and Statistical Manual of Mental Disorders* (Third or Fourth Edition) diagnostic criteria or *International Statistical Classification of Diseases, Tenth Revision* codes used in prior studies.^54^ The weighted CUD prevalence (3.7%) in the total sample of our study, which is representative of the VHA patients aged 65 to 80 years who met the eligibility criteria, was higher but still comparable with a study in veterans (mean [SD] age, 62.2 [15.7] years) in 2019 to 2020, which reported 2.7% of past 6-month CUD based on the Cannabis Use Disorder Identification Test–Revised (CUDIT-R).^48^ Another study in VHA patients (mean [SD] age, 57 [14.4] years) also showed similar CUD prevalence in 2019 while using clinician-based CUD diagnostic codes: 2.25% in states with nonlegal cannabis, followed by states with medical (2.54%) and recreational (2.56%) cannabis laws.^16^

The weighted prevalence of past-year cannabis use among the VHA patients in our study was higher compared with 2022 estimates for their counterparts 65 years or older in the civilian population: 14.1% vs 8.4%.^9^ However, the patterns and correlates of past 30-day cannabis use were consistent with findings in the general population.^3,42,76^ Smoking was the most common form of cannabis use in our sample, and use of edibles was higher in women vs men, similar to what has been observed in the general population of adults 60 years or older.^5^ Frequency of past 30-day cannabis use was independently associated with higher odds of any CUD, similar to prior research linking frequent cannabis use to increased CUD risk.^37,38^ Sociodemographic correlates of past 30-day cannabis use in older veterans were similar to those reported in past research of civilian populations^42,76^ and indicated socioeconomic disparities in cannabis use, which was higher among those who were younger (65-75 vs ≥76 years), unemployed or experiencing financial hardships, and unmarried or unpartnered. In line with previous studies in veterans 18 years or older,^43,44,45,77^ our findings showed higher cannabis use among respondents who resided in states with cannabis legalization (also in line with studies of non-VHA clinical populations^17^) and those reporting alcohol, tobacco, and illicit drug use, suggesting the need for cannabis use screening among veterans with these characteristics.

Compared with the general population, veterans are more likely to report mental health issues^53,78,79^ and substance use disorders^80^ and have higher suicide risk.^81^ It was expected that 1 of the top 3 reasons for lifetime medical cannabis use among the VHA patients in our study was management of mental health concerns, including anxiety, depression, and PTSD. Although anxiety was associated with higher odds of CUD, there were no differences by depression or PTSD status. This finding may reflect VHA patients’ greater access to evidence-based therapy and treatment options recommended by VHA clinicians as alternatives to cannabis use.^82^

Given rapid cannabis legalization affecting rates of use^1^ and CUD^16^ and increasing social acceptance of cannabis use,^10,18^ more focus is needed on negative health consequences of cannabis use in older veterans, such as higher risk for cardiovascular^31,32,33,34^ and respiratory health outcomes,^30^ neuropsychiatric disorders,^26,27,28,29^ and development of CUD.^36^ Older veterans may be at risk for THC intoxication, not tolerating cannabis potency that has increased in recent years^83,84,85^ or latent THC components found in products that are being marketed as cannabidiol only.^24^ Prevention of problematic cannabis use and CUD is also crucial in older veterans due to the elevated risk of suicide ideation^48^ and exacerbation of mental health concerns^86^ experienced by aging populations.

Findings from past research and our study highlight a potential need to include screening for cannabis use in clinical settings and to reinforce efforts to prevent CUD among veterans 65 years or older. Although the US Preventive Services Task Force^87^ recommends screening for any unhealthy substance use,^88^ the VA has opted not to engage in universal drug screening or screening for cannabis use at this juncture. The common and frequent cannabis use among older veterans in our study is concerning and suggests that this decision may need to be reconsidered. Past and emerging studies emphasize the importance of integrating cannabis screening and assessment for CUD in the primary care setting,^89,90,91^ which can be implemented via the Alcohol Substance Involvement Screening Test,^91^ Drug Abuse Screening Test 10,^92^ and Single-item Screen-Cannabis,^93^ with a follow-up assessment of CUD via *DSM-5*^57^ or Substance Use Symptom Checklist (a scaled measure of *DSM-5*)^90^ or CUDIT-R.^48^ Improved identification of patients at risk or with CUD can also help with linkage to care or referrals.^90,91^ Current VA guidelines recommend that patients with CUD be offered referral to mental health services for evidence-based treatments, including motivational interviews,^94^ contingency management,^95^ and cognitive behavioral therapy.^96^ However, if unidentified, patients cannot be offered existing evidence-based treatments. Despite increasing cannabis use among older adults, there is an inadequate evidence base on therapeutic benefits and potential harms from cannabis use among older people.^25,97^ Screening and informing older veterans about risks of cannabis use is critical because cannabis use may confer more health risks^25,26,27,28,29,30,31,32,33,34,98^ than benefits.^99,100,101^

### Limitations

This study has some limitations. First, this was a cross-sectional study, which precludes prospective examination of associations between patient characteristics and patterns of cannabis use. Second, this was an EHR-based cohort of US veterans 65 years or older; thus, results may not generalize to nonveteran or non-VHA populations^53^ or women. However, we oversampled women to ensure representation in the study. Third, these were self-reported data, provided via telephone interviews, regarding lifetime substance use behaviors; thus, recall and social desirability biases may have occurred. Responses about cannabis use could have varied by cannabis legalization status of the state of residence. It is possible that some participants did not accurately report their cannabis use. However, self-report is considered an accepted and valid method of assessing substance use used in many cohort studies^102,103^ and in national surveys.^3,5,76^ Furthermore, participants in this study were provided a National Institutes of Health Certificate of Confidentiality to reassure them that their data responses would remain private. Although self-administered online questionnaires can mitigate social-report biases for collection of sensitive data,^104,105^ telephone interviews used in the study may be a more effective method for data collection among older adults, as in our study.

## Conclusions

To our knowledge, this was the first large national study that used combined EHR and participant interview data to examine factors associated with current cannabis use and CUD among older veterans. Cannabis use was common and similar to estimates reported in younger populations; more than one-third of the participants with past 30-day use had any CUD. Anxiety, use of illicit drugs, frequent past 30-day cannabis use, and use of inhaled vs edible cannabis were associated with higher odds of any CUD. Given high rates of frequent cannabis use and CUD among older veterans, screening for cannabis use in clinical settings may be necessary, alongside referrals to appropriate specialty treatment.

## Notes

Supplement 1. **eFigure 1.** Cannabis use and any past 12-month cannabis use disorder among U.S. Veterans by gender: 2020-2023, n=4,503 **eFigure 2.** Forms of any lifetime cannabis use among U.S. Veterans 65 years and older: 2020-2023 **eFigure 3.** Reasons for any lifetime cannabis use among U.S. Veterans: 2020-2023, n=3,409 **eFigure 4.** Medical reasons for lifetime cannabis use among U.S. Veterans 65 years and older: n=4,503 **eFigure 5.** Any past 12-month cannabis use disorder among U.S. Veterans 65 years and older who report past 30-day cannabis use: 2020-2023, n=1,193 **eFigure 6.** Frequent current cannabis use and any past 12-month cannabis use disorder among U.S. Veterans 65 years and older who report past 30-day cannabis use: 2020-2023, n=1,193 **eTable 1.** Comparison of respondents and non-respondents among U.S. Veterans: 2020-2023 **eTable 2.** Survey items and measures for patterns of cannabis and tobacco use, and diagnostic criteria for cannabis use disorder among U.S. Veterans: 2020-2023 **eTable 3.** Medical reasons for cannabis use among U.S. Veterans: 2020-2023 **eTable 4.** Characteristics of those with versus without any past 12-month cannabis use disorder among U.S. Veterans reporting past 30-day cannabis use: 2020-2023, n=1,190 **eTable 5.** Association of forms and frequency of use with past 12-month CUD among U.S. Veterans who reported any past 30-day cannabis use: 2020-2023, n=1,193 (Sensitivity models)

Supplement 2.
Data Sharing Statement
