# Supplementary material for: Cannabis Use Among Older Adults
Source: JAMA Netw Open. 2025 May 14;8(5):e2510173. doi: 10.1001/jamanetworkopen.2025.10173 (PMC12079303; doi:10.1001/jamanetworkopen.2025.10173)
Supplement: Supplement 1. — eFigure 1. Cannabis use and any past 12-month cannabis use disorder among U.S. Veterans by gender: 2020-2023, n=4,503 eFigure 2. Forms of any lifetime cannabis use among U.S. Veterans 65 years and older: 2020-2023 eFigure 3. Reasons for any lifetime cannabis use among U.S. Veterans: 2020-2023, n=3,409 eFigure 4. Medical reasons for lifetime cannabis use among U.S. Veterans 65 years and older: n=4,503 eFigure 5. Any past 12-month cannabis use disorder among U.S. Veterans 65 years and older who report past 30-day cannabis use: 2020-2023, n=1,193 eFigure 6. Frequent current cannabis use and any past 12-month cannabis use disorder among U.S. Veterans 65 years and older who report past 30-day cannabis use: 2020-2023, n=1,193 eTable 1. Comparison of respondents and non-respondents among U.S. Veterans: 2020-2023 eTable 2. Survey items and measures for patterns of cannabis and tobacco use, and diagnostic criteria for cannabis use disorder among U.S. Veterans: 2020-2023 eTable 3. Medical reasons for cannabis use among U.S. Veterans: 2020-2023 eTable 4. Characteristics of those with versus without any past 12-month cannabis use disorder among U.S. Veterans reporting past 30-day cannabis use: 2020-2023, n=1,190 eTable 5. Association of forms and frequency of use with past 12-month CUD among U.S. Veterans who reported any past 30-day cannabis use: 2020-2023, n=1,193 (Sensitivity models) [file jamanetwopen-e2510173-s001.pdf]

# Supplemental Online Content

Pravosud V, Lum E, Vali M, et al. Cannabis use among older adults. *JAMA Netw Open*. 2025;8(5):e2510173. doi:10.1001/jamanetworkopen.2025.10173

**eFigure 1.** Cannabis use and any past 12-month cannabis use disorder among U.S. Veterans by gender: 2020-2023, n=4,503

**eFigure 2.** Forms of any lifetime cannabis use among U.S. Veterans 65 years and older: 2020-2023

**eFigure 3.** Reasons for any lifetime cannabis use among U.S. Veterans: 2020-2023, n=3,409

**eFigure 4.** Medical reasons for lifetime cannabis use among U.S. Veterans 65 years and older: n=4,503

**eFigure 5.** Any past 12-month cannabis use disorder among U.S. Veterans 65 years and older who report past 30-day cannabis use: 2020-2023, n=1,193

**eFigure 6.** Frequent current cannabis use and any past 12-month cannabis use disorder among U.S. Veterans 65 years and older who report past 30-day cannabis use: 2020-2023, n=1,193

**eTable 1.** Comparison of respondents and non-respondents among U.S. Veterans: 2020-2023

**eTable 2.** Survey items and measures for patterns of cannabis and tobacco use, and diagnostic criteria for cannabis use disorder among U.S. Veterans: 2020-2023

**eTable 3.** Medical reasons for cannabis use among U.S. Veterans: 2020-2023

**eTable 4.** Characteristics of those with versus without any past 12-month cannabis use disorder among U.S. Veterans reporting past 30-day cannabis use: 2020-2023, n=1,190

**eTable 5.** Association of forms and frequency of use with past 12-month CUD among U.S. Veterans who reported any past 30-day cannabis use: 2020-2023, n=1,193 (Sensitivity models)

This supplemental material has been provided by the authors to give readers additional information about their work.

© 2025 Pravosud V et al. *JAMA Network Open*.

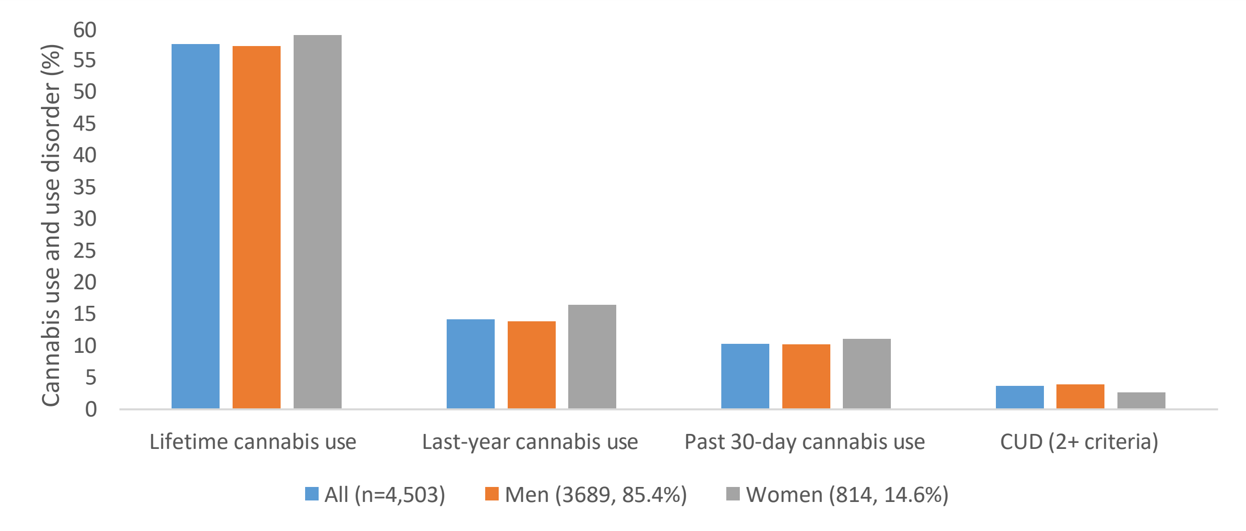


**eFigure 1. Cannabis use and any past 12-month cannabis use disorder among U.S. Veterans by gender: 2020-2023, n=4,503**

*Results shown are weighted percentages for endorsing cannabis use (smoking, vaping, dabbing, or edibles) among all respondents and by gender.*
*Cannabis use disorder (CUD) among those who reported past 30-day cannabis use was defined as reporting two or more of the following 11 criteria: tolerance; withdrawal/high use; taken in larger amounts or over a longer period than intended; a great deal of time getting using or recovering from use; important activities given up; recurrent psychological or physical problems; hazardous use; recurrent social problems; failure to fulfill obligations; craving; and/or persistent desire to cut down*

© 2025 Pravosud V et al. *JAMA Network Open*.

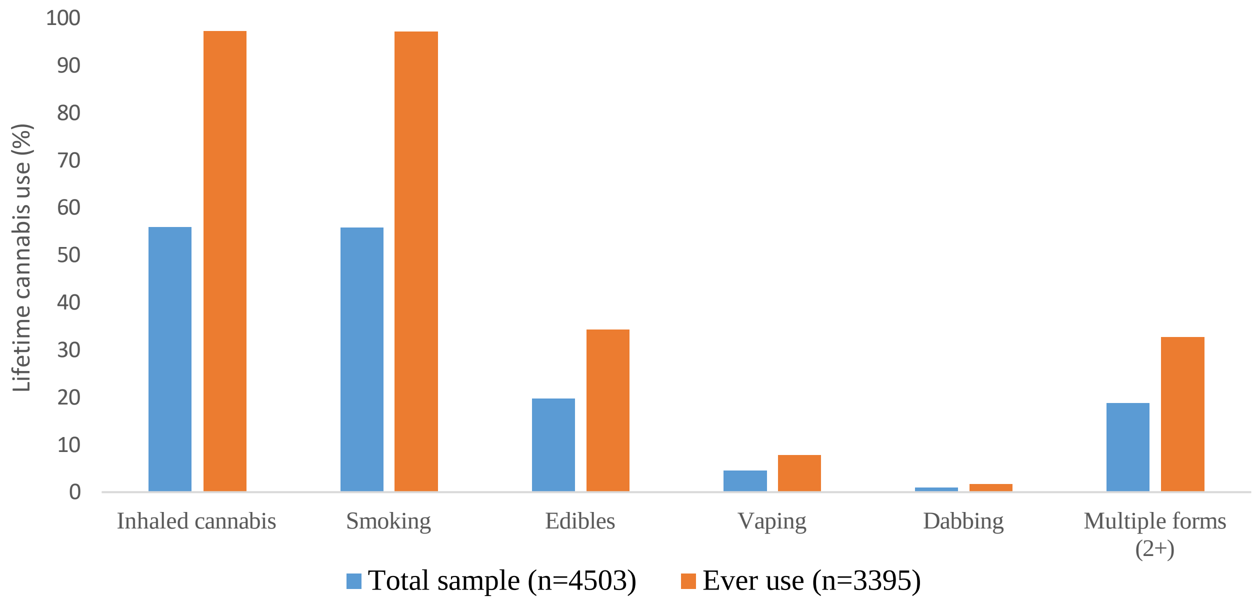


**eFigure 2. Forms of any lifetime cannabis use among U.S. Veterans 65 years and older: 2020-2023**

*Results shown are weighted percentages by modes of any lifetime cannabis use (smoking, vaping, dabbing, edibles).*
*Inhaled cannabis: any smoking, vaping, and/or dabbing.*
*Multiple forms: reporting any two or more forms of use, including smoking, vaping, dabbing, and/or edibles*

© 2025 Pravosud V et al. *JAMA Network Open*.

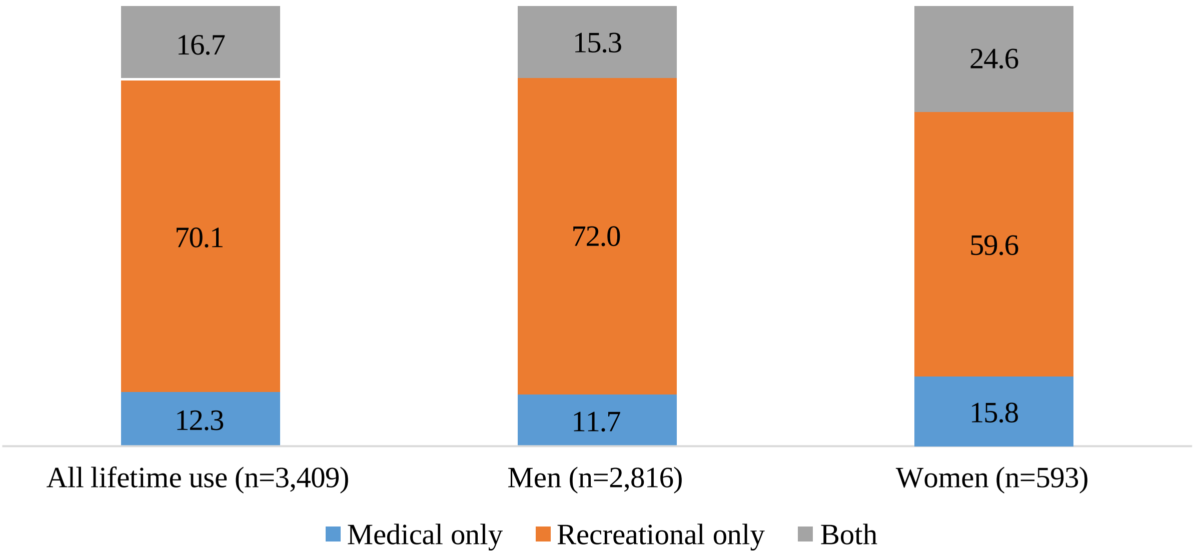


**eFigure 3. Reasons for any lifetime cannabis use among U.S. Veterans: 2020-2023, n=3,409**

*Results shown are weighted percentages among those with available data on forms of lifetime cannabis use (smoking, vaping, dabbing, use of edibles, or topicals) and by gender*

© 2025 Pravosud V et al. *JAMA Network Open*.

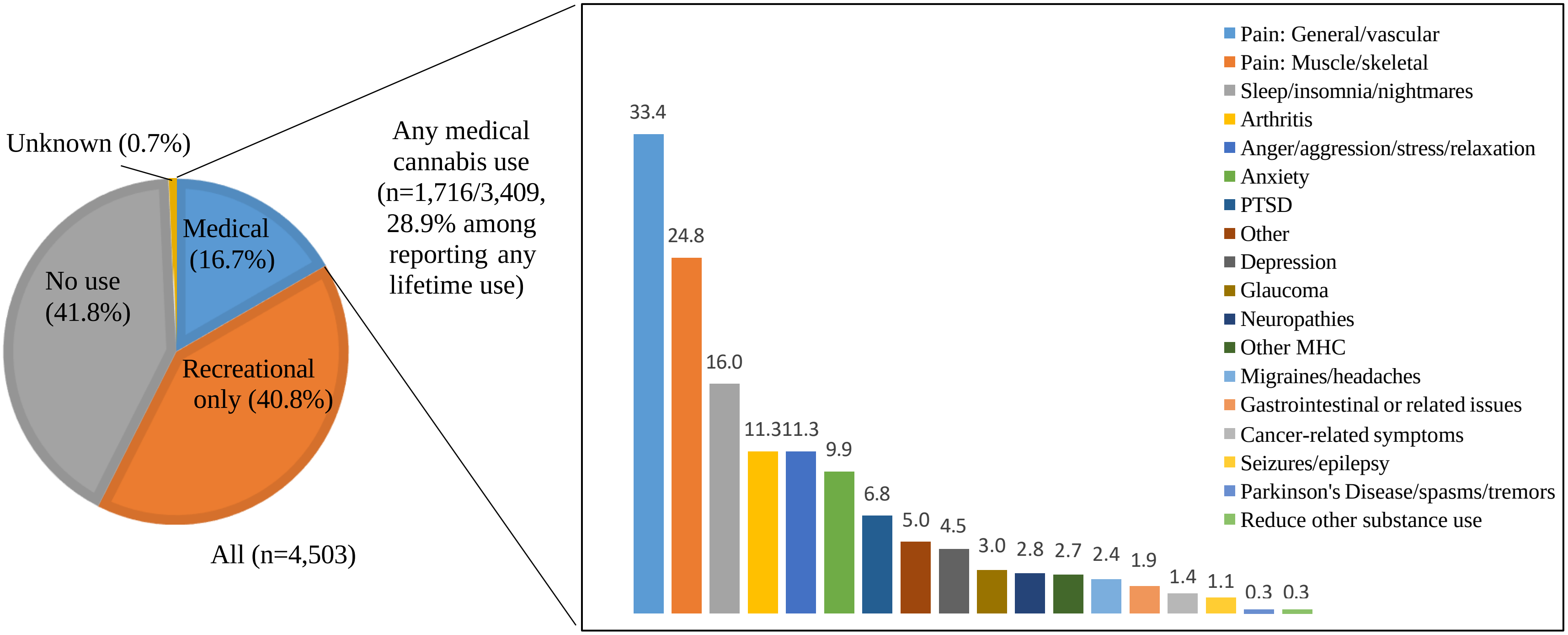


**eFigure 4. Medical reasons for lifetime cannabis use among U.S. Veterans 65 years and older: n=4,503**

*Results shown are weighted percentages for endorsing reasons for lifetime cannabis use: medical only, recreational only, or both.*
*Specific reasons for medical use (not mutually exclusive) were asked of those who reported lifetime cannabis use in any form (smoking, vaping, dabbing, edibles, or topicals) for any medical use (1,716 or 16.7%), including medical only (593, 7.1%) or medical and recreational purposes (1,123, 9.6%)*

© 2025 Pravosud V et al. *JAMA Network Open*.

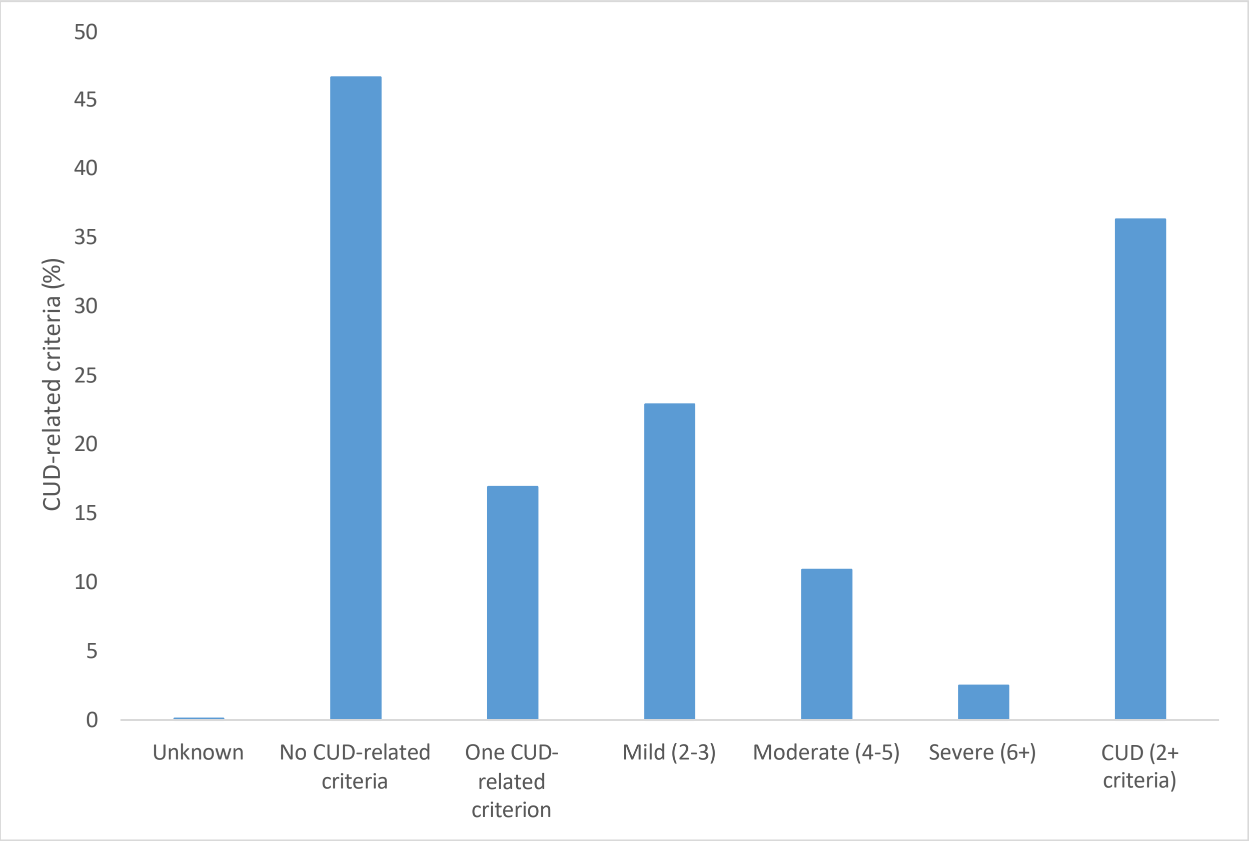


**eFigure 5. Any past 12-month cannabis use disorder among U.S. Veterans 65 years and older who report past 30-day cannabis use: 2020-2023, n=1,193**

*Results shown are weighted percentages for endorsing criteria related to cannabis use disorder (CUD). Cannabis use disorder (CUD) defined as reporting two or more of the following 11 criteria: tolerance; withdrawal/high use; taken in larger amounts or over a longer period than intended; a great deal of time getting using or recovering from use; important activities given up; recurrent psychological or physical problems; hazardous use; recurrent social problems; failure to fulfill obligations; craving; and/or persistent desire to cut down*

© 2025 Pravosud V et al. *JAMA Network Open*.

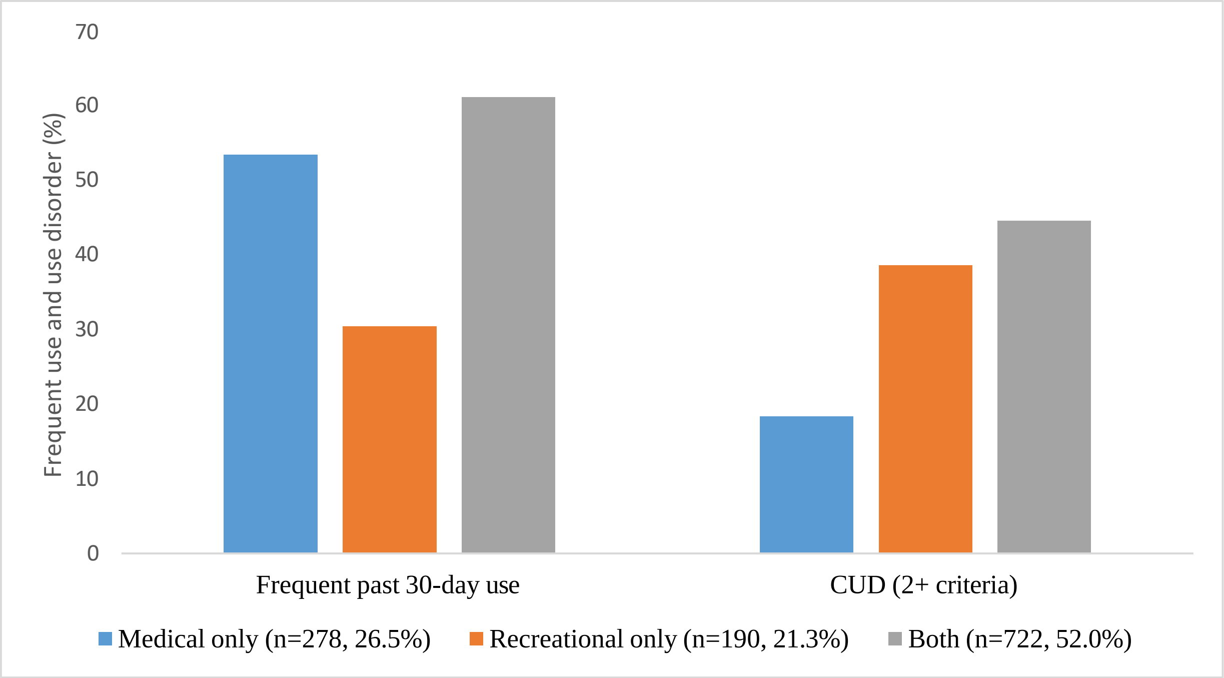


**eFigure 6. Frequent current cannabis use and any past 12-month cannabis use disorder among U.S. Veterans 65 years and older who report past 30-day cannabis use: 2020-2023, n=1,193**

*Results shown are weighted row percentages by reasons of lifetime cannabis use.*
*Cannabis use disorder (CUD) defined as reporting two or more of the following 11 criteria: tolerance; withdrawal/high use; taken in larger amounts or over a longer period than intended; a great deal of time getting using or recovering from use; important activities given up; recurrent psychological or physical problems; hazardous use; recurrent social problems; failure to fulfill obligations; craving; and/or persistent desire to cut down*

© 2025 Pravosud V et al. *JAMA Network Open*.

**eTable 1. Comparison of respondents and non-respondents among U.S. Veterans: 2020-2023**

| Characteristic | Respondents (n=4,503) | Non-respondents (n=4,409) | P[a] |
|---|---|---|---|
| **Age** | | | 0.04 |
| 65-70 | 2,474 (54.9) | 2,533 (57.5) | |
| 71-75 | 1,644 (36.5) | 1,541 (35.0) | |
| 76-80 | 385 (8.5) | 335 (7.6) | |
| **Race** | | | <.001 |
| American Indian or Alaska Native | 56 (1.2) | 49 (1.1) | |
| Asian | 35 (0.8) | 41 (0.9) | |
| Black or African American | 850 (18.9) | 794 (18.0) | |
| Native Hawaiian or Other Pacific Islander | 49 (1.1) | 53 (1.2) | |
| White | 3,409 (75.7) | 3,147 (71.4) | |
| Not defined[b] | 104 (2.3) | 325 (7.4) | |
| **Ethnicity** | | | <.001 |
| Hispanic or Latino | 219 (4.9) | 210 (4.8) | |
| Not Hispanic or Latino | 4,168 (92.6) | 3,942 (89.4) | |
| Unknown | 116 (2.6) | 257 (5.8) | |

*Results shown are frequencies and unweighted column percentages: n (%).*
[a]*P-values obtained from Pearson's Chi-squared tests.*
[b]*Included n=1 respondent with more than one race.*

© 2025 Pravosud V et al. *JAMA Network Open*.

**eTable 2. Survey items and measures for patterns of cannabis and tobacco use, and diagnostic criteria for cannabis use disorder among U.S. Veterans: 2020-2023**

| ***Cannabis use***[a] | **Question** | **Response options** |
|---|---|---|
| Lifetime cannabis use in any form (base) | Have you ever used marijuana in any form? | Yes; No |
| Lifetime cannabis use by forms (base) | Have you ever ________marijuana?<br>1) Smoked (e.g., a joint, pipe, bong, spliff, or blunt)<br>2) Vaporized<br>3) Dabbed<br>4) Used edible (e.g., brownies, gummy candies, tinctures, or sublingual drops)<br>5) Topically applied marijuana (e.g., lotions, balms, or oils) | Yes; No |
| Years of daily or near-daily cannabis smoking | Over the entire period you _______marijuana, how many years did you smoke marijuana on a daily or near-daily basis?<br>1) Smoked | ____years |
| Past 30-day cannabis use | Have you _______marijuana in the last 30 days? (base)<br>1) Smoked<br>2) Vaporized<br>3) Dabbed<br>4) Used edible<br>5) Topically applied | Yes; No |
| | For smoking cannabis only: In the last 30 days, have you smoked a_________(base) | Joint; pipe; bong; spliff; blunt; Refused; Don't know |
| | In the last 30 days, how many days per week did you ____?<br>1) Smoke a (joint; pipe; bong; spliff; blunt)<br>2) Vaporize<br>3) Dab<br>4) Use edible marijuana<br>5) Topically apply marijuana | Every day; 6 days/week; 5 days/week; 4 days/week; 3 days/week; 2 days/week; 1 day/week; #days/month |
| Past-year cannabis use | Have you ______marijuana in the last year?<br>1) Smoked (such as a joint, pipe, bong, spliff, or blunt)<br>2) Vaped<br>3) Dabbed<br>4) Used edible<br>5) Topically applied | Yes; No |
| ***Tobacco use***[b] | | |
| Lifetime cigarette use | Have you ever used tobacco in any form, including vaping nicotine? | Yes; No |
| | Have you smoked more than 100 cigarettes in your lifetime? | Yes; No |
| | When did you last smoke a cigarette? | days ago; weeks ago; __months ago; __years ago; ___Year last used__; Age last used |
| Lifetime use of other tobacco products | Have you ever smoked a ________?<br>1) Cigarillo<br>2) Cigarette<br>3) Cigarillo<br>4) Cigars<br>5) Pipe<br>6) E-cigarette (electronic cigarette) | Yes; No |

© 2025 Pravosud V et al. *JAMA Network Open*.

| | | |
|---|---|---|
| | Which category best describes the total number of times of ______you've smoked [or vaporized nicotine] over your lifetime?<br>1) Cigarillos<br>2) Cigarettes<br>3) Cigarillos<br>4) Cigars<br>5) Pipes<br>6) E-cigarettes | 51-500; 501-1000 |
| Past 30-day tobacco use | In the last 30 days, how many days per week did you smoke a_______?<br>1) Cigarette<br>2) Cigarillo<br>3) Cigars<br>4) Pipe<br>5) E-cigarette | Every day; 6 days/week; 5 days/week; 4 days/week; 3 days/week; 2 days/week; 1 day/week; # days/month |
| **Past 12-month cannabis use disorder (CUD)** | **Diagnostic criteria** | **Definition used in the analysis** |
| CUD | 1) Tolerance,<br>2) Withdrawal/high use,<br>3) Taken in larger amounts or over a longer period than intended,<br>4) A great deal of time getting using or recovering from use,<br>5) Important activities given up,<br>6) Recurrent psychological or physical problems,<br>7) Hazardous use,<br>8) Recurrent social problems,<br>9) Failure to fulfill obligations,<br>10) Craving,<br>11) Persistent desire to cut down | Reporting 2 or more of the diagnostic criteria |

[a]*– Survey items on lifetime cannabis use were the base questions for past 30-day and past-year use, and reasons for use. All forms of cannabis use were reported. For the modeling, (frequent) current cannabis use was defined as smoking, vaping, dabbing, or use of edibles in the past 30 days.*
[b]*– Current tobacco use was defined as reporting tobacco smoking (cigarette, cigarillo, cigar, pipe, or use of electronic cigarettes, e-cigarettes) at least 16 days in the past 30 days. Tobacco cigarette smoking frequency was assessed among those who reported having smoked >100 cigarettes in the lifetime. Other tobacco product use (cigarillos, cigars, pipes, or e-cigarettes) frequency was assessed among those reporting use of at least 51 products in the lifetime. Thus, no current use (i.e., past) tobacco use status was defined as no past 30-day use among those who reported ever smoking >100 cigarettes or using >51 of other tobacco products.*
*All survey questions included "Refused" and "Don't know" response options*

© 2025 Pravosud V et al. *JAMA Network Open*.

**eTable 3. Medical reasons for cannabis use among U.S. Veterans: 2020-2023**

| ***Analyzed categories*** | ***What medical reasons have you used it for?*** |
|---|---|
| (1) Anxiety | 1. Anxiety |
| (2) Arthritis | 2. Arthritis |
| (3) Cancer-related symptoms | 3-5. Cancer: general, pain, nausea and vomiting |
| (4) Depression | 6. Depression; |
| (5) Gastrointestinal or related issues | 7-8. Appetite; nausea/vomiting; |
| (6) Glaucoma or vision problems | 9. Glaucoma |
| (7) Migraines, headaches | 10. Migraines/headaches |
| (8) Neuropathic pain | 11. Neuropathies |
| (9) General or vascular pain | 12-14. Pain: general, vascular; spinal cord damage |
| (10) Muscle or skeletal pain | 15. Pain: muscle/skeletal |
| (11) Parkinson's disease, spasms, tremors | 16-17. Parkinson’s Disease; spasms/tremors |
| (12) Post-traumatic stress disorder (PTSD) | 18. PTSD |
| (13) Reduction of other substance use | 19. Opiate therapy (in lieu of) |
| (14) Seizures or epilepsy | 20. Seizures/epilepsy |
| (15) Sleep difficulties | 21-22. Sleep/insomnia; nightmares/night terrors |
| (16) Other mental health issues | 23. Bipolar disorder |
| (17) Other clinical conditions or medical concerns | 24-34. Crohn’s Disease; diabetes/high blood sugar; hepatitis-C; HIV/AIDS; Huntington’s Disease; hypertension/high blood pressure; IBS/Stomach issues; libido; lung-related issues; multiple sclerosis; psoriasis, and other reported clinical conditions not listed in the survey |
| Anger, aggression, relaxation, or stress | 35-37. Anger/aggression; relaxation; stress |
| ***Options (survey items) not reported by respondents*** | 38-42. Allergies; ALS/Lou Gehrig’s (Amyotrophic Lateral Sclerosis); Alzheimer’s/dementia; Sickle cell anemia; Terminal illness |

*[*] Respondents could select among the 42 options included in the survey or choose the “Other” category and provide information on other conditions not included in the questionnaire. Because of sparce data or small cell sizes for some medical reasons, we created 17 combined categories. We also reported percentages of those who used cannabis for management of anger, aggression, relaxation, or stress.*

© 2025 Pravosud V et al. *JAMA Network Open*.

**eTable 4. Characteristics of those with versus without any past 12-month cannabis use disorder among U.S. Veterans reporting past 30-day cannabis use: 2020-2023, n=1,190**

| Characteristic | CUD[a] (546, 36.3%) | No CUD (644, 63.7%) | AOR (95% CI)[b] | P[b] |
|---|---|---|---|---|
| **Age** | | | | |
| 65-70 | 263 (43.2, [34.1-52.3]) | 295 (31.9, [24.4-39.4]) | 6.03 (2.26-16.12) | <.001 |
| 71-75 | 241 (52.1, [42.8-61.4]) | 277 (47.5, [38.0-57.0]) | 4.86 (1.89-12.48) | .001 |
| 76-84 | 42 (4.7, [3.1-6.3]) | 72 (20.6, [10.6-30.6]) | Reference | |
| **Gender** | | | | |
| Men | 490 (89.3, [83.3-95.3]) | 525 (81.5, [74.8-88.1]) | 3.42 (1.69-6.93) | <.001 |
| Women | 56 (10.7, [4.7-16.7]) | 119 (18.5, [11.9-25.2]) | Reference | |
| **Race and ethnicity** | | | | |
| Black or African American and non-Hispanic | 123 (22.3, [14.8-29.8]) | 135 (15.5, [9.7-21.4]) | 1.33 (0.70-2.53) | .38 |
| Hispanic | 26 (4.1, [1.1-7.1]) | 26 (5.2, [0.8-9.5]) | 1.02 (0.38-2.70) | .98 |
| White and non-Hispanic | 373 (71.1, [63.1-79.1]) | 461 (75.2, [67.6-82.7]) | Reference | |
| Other race and non-Hispanic[c] | 24 (2.5, [1.4-3.6]) | 22 (4.2, [0.6-7.7]) | 0.68 (0.25-1.83) | .44 |
| **Marital status** | | | | |
| Other | 324 (53.8, [44.2-63.3]) | 342 (56.2, [46.8-65.6]) | 0.73 (0.41-1.31) | .29 |
| Married, partner, engaged | 222 (46.2, [36.7-55.8]) | 302 (43.8, [34.4-53.2]) | Reference | |
| **Education** | | | | |
| Bachelor's and beyond | 103 (23.8, [15.2-32.5]) | 177 (29.2, [20.4-37.9]) | Reference | |
| High school/Some College degree | 414 (71.9, [63.1-80.7]) | 444 (68.7, [59.9-77.5]) | 1.16 (0.64-2.12) | .62 |
| Less than high school graduate | 29 (4.3, [1.4-7.1]) | 23 (2.2, [0.4-3.9]) | 1.65 (0.48-5.71) | .43 |
| **Employment** | | | | |
| Employed | 36 (7.6, [1.9-13.2]) | 55 (7.6, [3.0-12.1]) | 0.68 (0.21-2.23) | .52 |
| Retired | 350 (65.6, [56.8-74.4]) | 418 (68.1, [59.5-76.7]) | Reference | |
| Other | 160 (26.8, [19.0-34.7]) | 171 (24.3, [16.4-32.3]) | 0.96 (0.54-1.71) | .89 |
| **Hard to pay for basic needs** (n=1,188) | 192 (27.7, [20.2-35.1]) | 154 (29.5, [20.2-38.7]) | 0.90 (0.50-1.63) | .73 |
| Not very hard | 352 (72.1, [64.6-79.6]) | 490 (70.5, [61.3-79.8]) | Reference | |
| **State cannabis legalization status** | | | | |

© 2025 Pravosud V et al. *JAMA Network Open*.

| | | | | |
|---|---|---|---|---|
| Non-legal | 138 (23.2, [16.1-30.2]) | 161 (24.7, [17.1-32.3]) | Reference | |
| Medical only | 183 (34.4, [25.3-43.5]) | 206 (32.9, [23.9-41.9]) | 1.38 (0.70-2.72) | .35 |
| Recreational+medical | 225 (42.5, [32.9-52]) | 277 (42.4, [23.9-41.9]) | 1.66 (0.89-3.09) | .11 |
| **Frequent past 30-day cannabis use:** ≥20 days | 392 (64.9, [55.5-74.4]) | 360 (45.4, [35.9-54.8]) | 2.64 (1.58-4.40) | <.001 |
| Non-frequent past 30-day use | 154 (35.1, [25.6-44.5]) | 284 (54.6, [45.2-64.1]) | Reference | |
| **Reasons for lifetime cannabis use** (n=1,189) | | | | |
| Medical only | 80 (13.4, [7.2-19.5]) | 198 (34.1, [24.7-43.5]) | 0.27 (0.14-0.53) | <.001 |
| Recreational only or both | 465 (86.5, [80.3-92.7]) | 446 (65.9, [56.5-75.3]) | Reference | |
| **Any past 30-day tobacco use**[d] | | | | |
| Never | 66 (11.9, [5.8-18.1]) | 105 (22.6, [14.1-31.0]) | Reference | |
| No current use | 321 (58.9, [49.6-68.2]) | 351 (59.8, [50.7-68.9]) | 1.41 (0.66-3.00) | .38 |
| Current (>16 days/ past month) | 159 (29.2, [20.6-37.8]) | 188 (17.6, [12.1-23.2]) | 1.64 (0.66-4.07) | .28 |
| **AUDIT-C** (Positive vs Negative; n=1189) | 207 (37.0, [28.0-46.0]) | 208 (29.7, [21.4-38.0]) | 1.10 (0.66-1.86) | .71 |
| **Past 30-day illicit drug use** (n=1182)[e] | 22 (2.2, [1.2-3.2]) | 10 (0.6, [0.2-1.0]) | 2.69 (1.06-6.85) | .04 |
| **≥10 symptoms based on the Generalized Anxiety Disorder screener, GAD-7** (n=1,189) | 105 (19.6, [12-27.3]) | 81 (8.1, [2.9-13.3]) | 5.01 (2.06-12.14) | <.001 |
| **≥10 depressive symptoms based on the Patient Health Questionnaire, PHQ-8** (n=1,188) | 145 (20.1, [13.7-26.5]) | 105 (16.0, [8.8-23.1]) | 0.89 (0.44-1.80) | .75 |
| **≥19 based on PCL-8 inventory** (n=1,185) | 57 (6.0, [4.2-7.9]) | 41 (5.3, [1.6-8.9]) | 0.44 (0.17-1.17) | .099 |
| **Loneliness** (n=1,188) | | | | |
| Not lonely | 352 (76.0, [69.9-82.0]) | 495 (78.0, [70.0-85.9]) | Reference | |
| Lonely | 192 (23.8, [17.8-29.8]) | 149 (22.0, [14.1-30.0]) | 1.22 (0.67-2.22) | .50 |
| **CAN score** (n=1,187) | | | | |
| <50 | 136 (30.3, [20.9-39.7]) | 171 (26.3, [18.8-33.8]) | Reference | |
| 50 to <75 | 252 (44.0, [34.7-53.4]) | 285 (41.9, [32.6-51.2]) | 0.99 (0.53-1.85) | .96 |
| ≥75 | 156 (25.5, [17.8-33.2]) | 187 (31.7, [22.1-41.4]) | 0.69 (0.35-1.37) | .29 |
| **≥1 deficit in activities of daily life support** (n=1,184) | 211 (36.2, [27.3-45.0]) | 197 (30.1, [21.3-38.9]) | 1.76 (1.05-2.95) | .03 |

© 2025 Pravosud V et al. *JAMA Network Open*.

| | | | | |
|---|---|---|---|---|
| **Falls in the past 12 months** (n=1,189) | 193 (29.0, [21.0-37.1]) | 226 (29.4, [21.4-37.3]) | 0.79 (0.45-1.40) | .42 |

*Results shown are frequencies and weighted percentages together with the 95% confidence intervals [%-%] among those reporting past 30-day cannabis use and non-missing data on cannabis use disorder (CUD; n=1,190), unless otherwise specified: n (%, [%-%]).*

*a – CUD was defined as reporting two or more of the following 11 criteria: tolerance; withdrawal/high use; taken in larger amounts or over a longer period than intended; a great deal of time getting using or recovering from use; important activities given up; recurrent psychological or physical problems; hazardous use; recurrent social problems; failure to fulfill obligations; craving; and/or persistent desire to cut down.*

*b – Results were obtained from an adjusted logistic regression with all listed variables entered simultaneously; CUD was the outcome variable: n= 1,164 observations used (miss=29, 2.4%), n=532 with CUD versus n=632 without CUD.*

*c – Other race and non-Hispanic category included respondents of another race (Asian: n=7, 0.7%, American Indian or Alaska Native: n=10, 0.4%, and Native Hawaiian or Other Pacific Islander: n=15, 0.6%), of multiple races (n=1, 0.04%), or of unknown or not reported race or ethnicity (n=13, 1.8%).*

*d – Smoking cigarette, cigarillo, cigars, pipe, or e-cigarette use >16 days in the past month.*

*e – Including cocaine, heroin, amphetamines, or other illicit drugs.*

*Abbreviations: CUD: Cannabis use disorder; AOR: Adjusted Odds Ratio, CI: confidence interval; PCL: posttraumatic stress disorder checklist for DSM-5; AUDIT-C: the Alcohol use Disorders Identification Test-Consumption; CAN score: the Care Assessment Need score*

© 2025 Pravosud V et al. *JAMA Network Open*.

**eTable 5. Association of forms and frequency of use with past 12-month CUD among U.S. Veterans who reported any past 30-day cannabis use: 2020-2023, n=1,193 (Sensitivity models)**

| | Outcome 1 (≥2 criteria) | | | | | | Outcome 2 (≥4 criteria) | | | |
|---|---|---|---|---|---|---|---|---|---|---|
| | **Model 1: CUD vs no CUD** | | **Model 2: CUD vs no CUD** | | **Model 3: CUD vs no CUD** | | **Model 4: CUD vs no CUD** | | **Model 5: CUD vs no CUD** | |
| **Forms of use & Covariates** | AOR (95% CI) | P | AOR (95% CI) | P | AOR (95% CI) | P | AOR (95% CI) | P | AOR (95% CI) | P |
| **Any smoking** (986; 72.4, [65.4-79.3]) | 2.99 (1.23-7.26) | .02 | | | | | 3.88 (1.08-14.02) | .04 | | |
| **Any vaping** (170; 11.5, [7.7-15.3]) | 1.56 (0.59-4.09) | .37 | | | | | 0.99 (0.39-2.51) | .98 | | |
| **Any dabbing** (32; 1.7, [0.6-2.8]) | 1.48 (0.26-8.53) | .66 | | | | | 1.32 (0.38-4.57) | .66 | | |
| **Any edibles** (339; 36.9, [29.8-43.9]) | 1.17 (0.57-2.39) | .67 | NA | NA | NA | NA | 1.15 (0.45-2.97) | .77 | NA | NA |
| **Frequent past 30-day use** (753; 52.4, [45.4-59.4]) | | | | | 2.02 (1.16-3.51) | .01 | 1.87 (0.89-3.90) | .10 | 1.79 (0.83-3.86) | .14 |
| **Form of use** | | | | | | | NA | NA | | |
| Edibles only (135; 21.7, [14.9-28.5]) | NA | NA | 0.21 (0.07-0.64) | .006 | 0.28 (0.09-0.89) | .03 | NA | NA | Reference | |
| Any inhaled use (1058; 78.3, [71.5-85.1]) | | | Reference | | Reference | | NA | NA | 14.85 (4.94-44.62) | <.001 |
| **Age** | | | | | | | | | | |
| 65-70 (558; 36.0, [30.0-41.9]) | 4.53 (1.91-10.72) | <.001 | 4.64 (1.94-11.14) | <.001 | 5.49 (2.15-14.02) | <.001 | 3.43 (1.33-8.87) | .01 | 3.47 (1.33-9.01) | .01 |
| 71-75 (521; 49.2, [42.2-56.2]) | 4.13 (1.70-10.05) | .002 | 4.26 (1.72-10.51) | .002 | 4.96 (1.89-13.03) | .001 | 3.18 (1.17-8.61) | .02 | 3.16 (1.14-8.80) | .03 |
| 76-84 (114; 14.8, [8.1-21.6]) | Reference | | Reference | | Reference | | Reference | | Reference | |
| **Gender** | | | | | | | | | | |
| Men (1018; 84.3, [79.6-89.1]) | 1.74 (0.76-3.99) | .19 | 1.50 (0.67-3.33) | .32 | 1.63 (0.71-3.72) | .25 | 1.41 (0.60-3.32) | .43 | 1.27 (0.53-3.06) | .60 |
| Women (175; 15.7, [10.9-20.4]) | Reference | | Reference | | Reference | | Reference | | Reference | |
| **Reasons for lifetime cannabis use** | | | | | | | | | | |
| Medical only (278, 26.5, [19.9-33.2]) | 0.39 (0.20-0.79) | .009 | 0.40 (0.2-0.79) | .009 | 0.36 (0.18-0.73) | .005 | 0.76 (0.29-1.98) | .58 | 0.8 (0.31-2.08) | .65 |
| Recreational only or both (912, 73.4, [66.7-80]) | Reference | | Reference | | Reference | | Reference | | Reference | |

*Numbers shown are frequencies, weighted row percentages together with the 95% confidence intervals for cannabis use disorder (CUD) by forms of use (not mutually exclusive): n (%; [%-%]).*
*Adjusted odds ratios for forms of use are shown in associations with two outcome variables – CUD defined as reporting any two (outcome 1) or four (outcome 2) of the following 11 criteria: tolerance; withdrawal; cannabis taken in larger amounts or over a longer period than intended; a great deal of time getting using or recovering from use; important activities given up; recurrent psychological or physical problems; hazardous use; recurrent social problems; failure to fulfill obligations; craving; and/or persistent desire to cut down.*
*Frequent past 30-day cannabis use was defined as smoking, vaping, dabbing, or using edibles 20 or more days in the past 30 days.*
*Leastwise deletion was used in all weighted models:*

© 2025 Pravosud V et al. *JAMA Network Open*.

*Model 1 – Variables: reporting either smoking, vaping, dabbing, or use of edibles: are primary independent variables, while adjusting for age, gender, and reasons of lifetime cannabis use (n=1182: 545 with CUD vs 637 without CUD).*
*Model 2 – Variables: a 2-level variable indicating forms of cannabis use: edibles only compared to any inhaled cannabis use, while adjusting for age, gender, and reasons of lifetime cannabis use (n=1189: 545 with CUD vs 644 without CUD).*
*Model 3 – Variables: a 2-level variable indicating forms of cannabis use: edibles only compared to any inhaled products, while adjusting for age, gender, frequency of past 30-day cannabis use, and reasons of lifetime cannabis use (n=1189: 545 with CUD vs 644 without CUD); reciprocal results for edibles only vs. any inhaled cannabis.*
*Model 4 – Variables: reporting either smoking, vaping, dabbing, or use of edibles: are primary independent variables, while adjusting for age, gender, and reasons of lifetime cannabis use (n=1182: 209 with CUD vs 973 without CUD).*
*Model 5 – Variables: a 2-level variable indicating forms of cannabis use: edibles only compared to any inhaled cannabis use, while adjusting for age, gender, and reasons of lifetime cannabis use (n=1189: 209 with CUD vs 980 without CUD).*
*Abbreviations: CUD: Cannabis use disorder; AOR: Adjusted odds ratio; CI: Confidence interval; NA: Not applicable*

© 2025 Pravosud V et al. *JAMA Network Open*.
