# Supplementary material for: Cannabis Use Among Older Adults
Source: JAMA Netw Open. 2025 May 14;8(5):e2510173. doi: 10.1001/jamanetworkopen.2025.10173 (PMC12079303; doi:10.1001/jamanetworkopen.2025.10173)
Supplement: Supplement 2. — Data Sharing Statement [file jamanetwopen-e2510173-s002.pdf]

Data Sharing Statement

Pravosud. Cannabis Use Among Older Adults. *JAMA Netw Open*. Published May 14, 2025. doi:10.1001/jamanetworkopen.2025.10173

Data
Data available: Yes
Data types: Other (please specify)
Additional Information: Access to the data is possible with adherence to VA policy and procedures. De-identified data may be made available upon reasonable request to the Principal Investigator, Salomeh Keyhani.
How to access data: Salomeh.Keyhani@va.gov
When available: With publication

Supporting Documents
Document types: Other (please specify)
Additional Information: NA
How to access documents: NA
When available: With publication

Additional Information
Who can access the data: Access to the data is possible with adherence to VA policy and procedures. De-identified data may be made available upon reasonable request to the Principal Investigator, Salomeh Keyhani.
Types of analyses: NA
Mechanisms of data availability: Access to the data is possible with adherence to VA policy and procedures. De-identified data may be made available upon reasonable request to the Principal Investigator, Salomeh Keyhani.
